# Advances in Pd-catalyzed C–C bond formation in carbohydrates and their applications in the synthesis of natural products and medicinally relevant molecules

**DOI:** 10.1039/d1ra06351k

**Published:** 2021-10-22

**Authors:** Nazar Hussain, Altaf Hussain

**Affiliations:** Department of Medicinal Chemistry, Faculty of Ayurveda, Institute of Medical Sciences, BHU Varanasi-221005 India nazar.hussain10@gmail.com nazar10@bhu.ac.in nazar.hussain@iaf.inrs.ca; Department of Chemistry, Govt. Degree College Poonch J&K India 185101

## Abstract

Advances in the Pd-catalyzed synthesis of *C*-glycosides and branched sugars are summarized herein and the strategies are categorized based on named reactions or types of sugar moieties involved in the reactions. These include cross-coupling reactions, C–H activations, and carbonylative cross-coupling reactions. Applications of Pd-catalyzed *C*-glycosylation reactions are discussed in the synthesis of natural products and biologically active molecules such as bergenin, papulacandin D, and SGLT2-inhibitors. Important mechanistic cycles are drawn and the mechanisms for how Pd-activates the sugar moieties for various coupling partners are discussed. The directing group-assisted *C*-glycosylation and some intramolecular C–H activation reactions are also included.

## Abstract 2

This review highlights the recent progress of Pd-catalyzed reactions in carbohydrates. Different types strategies and applications in the synthesis of natural products, SGLT2 inhibitors and biologically active have been included in this review.

## Graphical abstract

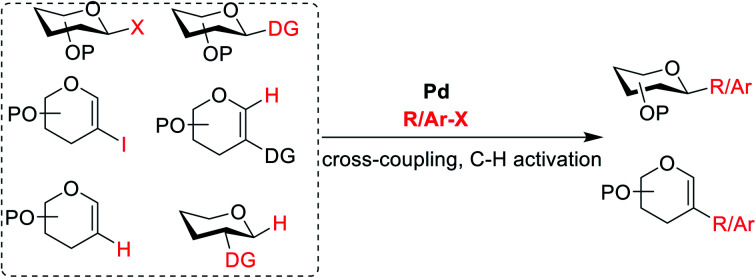

## 1. Introduction

Transition-metal-catalyzed cross-coupling reactions have emerged among the most widely used approaches in modern synthetic organic chemistry.^1–3^ The TM-catalyzed construction of structurally simple to complex molecules and natural products from readily available starting materials has wide applicability due to the low cost, low toxicity, and exceptional synthetic versatility of TMs. This approach allows the researcher to construct a variety of molecules *via* C–C, C-heteroatom linkages for both synthetic and industrial applications such as the synthesis of natural products, agrochemicals, and pharmaceuticals. Many catalytic cycles have been developed in the recent past using different types of transition metals and are applicable for the conversion of all types of organic molecules such as preactivated substrates and substrates without any preactivation, *i.e.*, C–H activation. In the beginning, these transition metal-catalyzed approaches relied on the use of pre-functionalized substrates, for example, in the Negishi (Zn), Stille (Sn), Kumada (Mg), Fukuyama (Zn), and Suzuki (B) reactions.^4,5^ More recently, C–C bond formation without the prefunctionalization of substrates, *i.e.*, C–H activation, has emerged as a more environmentally friendly, atomic economic strategy for linking molecules *via* the C–C bond.^6–9^ Considering the higher abundance of first-row transition metals such as Fe, Co, Cu, and Ni, their low prices, little or no toxicity, and unique catalytic characteristics, these metals have become substantially more attractive for cross-coupling reactions. Despite the curiosity for the utilization of first-row transition metals in cross-coupling reactions, the palladium (Pd)-catalyzed transformation of organic compounds *via* C–C and C-heteroatom in recent decades and has been extensively studied.^6–9^ The importance and success of Pd-catalyzed cross-coupling reactions are reflected in the Nobel Prize in Chemistry being awarded to Richard F. Heck, Eiichi Negishi, and Akira Suzuki in 2010.

**Fig. 1:**
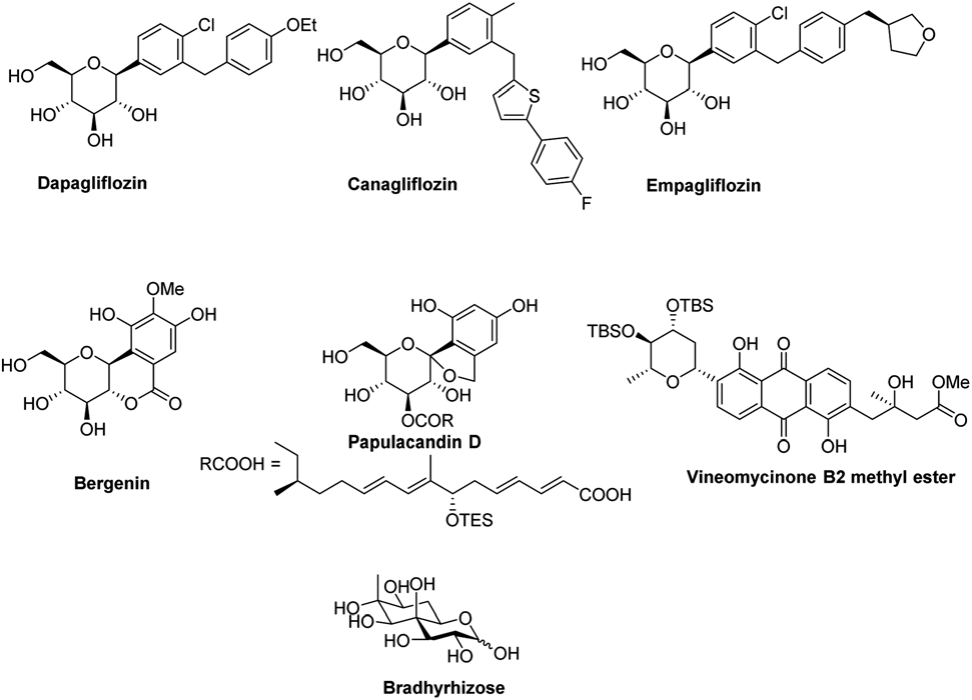
Some biologically active molecules involved in the Pd-catalyzed cross-coupling reactions.

**Fig. 2:**
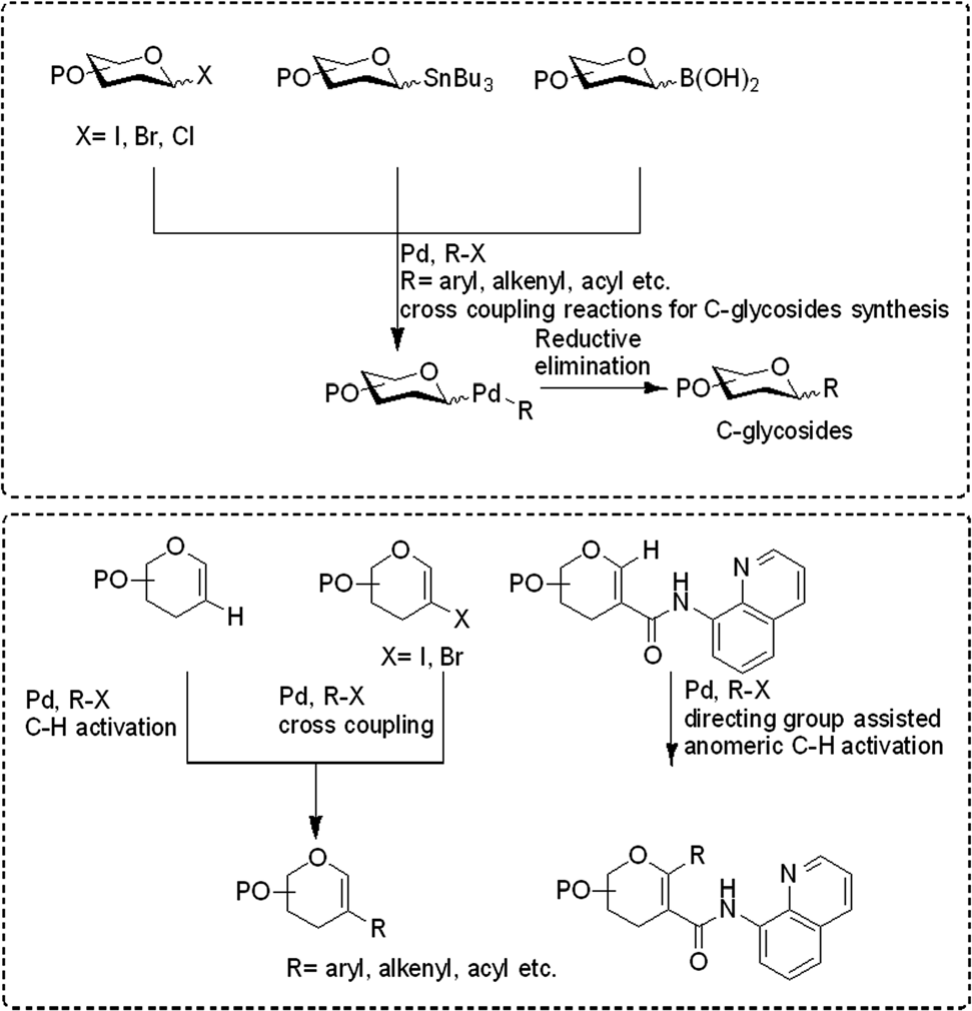
Coupling partners used in Pd catalyzed cross-coupling reactions for the synthesis of *C*-glycosides and *C*-branched sugars.

In the last 10 years, a handful of efficient protocols and novel methodologies have been developed for the synthesis of *C*-glycosides and branched sugars *via* Pd-catalyzed cross-coupling reactions.^10^*C*-Glycosides are ubiquitous structural motifs present in a wide range of natural products and commercially available drug molecules. Due to the stability of their C–C bonds towards enzymatic hydrolysis, *C*-glycosides have been exclusively used and synthesized as an artificial surrogate and mimic of *O-*glycosides.^11–15^ The importance of synthetic *C*-glycosides is well recognized due to their emergence as inhibitors such as flozin analogues, and have been used for the treatment of type 2 diabetes.^16–20^ Similarly, *C*-branched sugars have attracted great attention from synthetic organic chemists due to their importance as potential antibiotics^21^ and mimics of 2-*N*-acetyl sugars for cell surface engineering and as inhibitors for the biosynthesis of lipids.^22^ In order to access both *C*-glycosides and *C*-branched sugars, various types of strategies have been developed in the recent past but at the same time, a strong development has been made in the area of the Pd-catalyzed cross-coupling reaction of carbohydrates with different coupling partners. Pd-catalyzed cross-coupling reactions have great potential and have been extended for the synthesis of certain biologically potent molecules and marketed drugs such as flozin analogs, bergenin, papulacandin D, vineomycinone B2 methyl ester, bradyrhizose, and many others (Fig. 1). To obtain the *C*-glycosides and *C*-branched sugars, different types of sugar-derived coupling partners have been used, some of which are listed in Fig. 2. Although many reviews have already covered the chemical synthesis of *C*-glycosides^10,23–25^ and branched sugars,^26^ no advancement has been made that completely focuses on Pd-catalyzed reactions in carbohydrates. Considering the development made in the area of Pd catalysis in carbohydrates, we have tried to present a complete overhaul of such reactions in carbohydrates. In this review, we have categories the carbohydrate functionalization under Pd catalysis based on substrates and types of reactions under the following headings:

(1) *C*-Glycosylation *via* cross-coupling reactions. (2) Pd-catalyzed 2*C*-branched sugar synthesis.

## 2. *C*-Glycosylation *via* cross-coupling reactions

### 2.1. Suzuki–Miyaura cross-coupling approach for *C*-glycosides synthesis

**Scheme 1:**
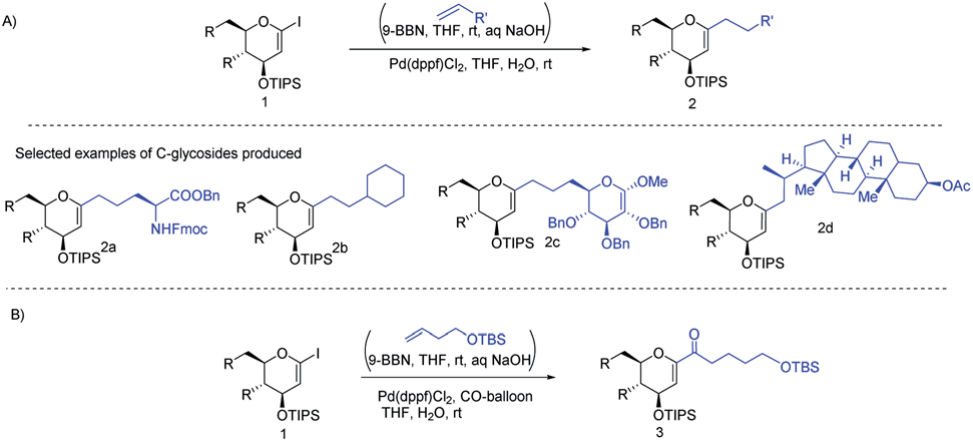
(A) Alkyl-*C*-glycals (B) acyl-*C*-glycals *via* the Suzuki–Miyaura cross-coupling approach.

**Scheme 2:**
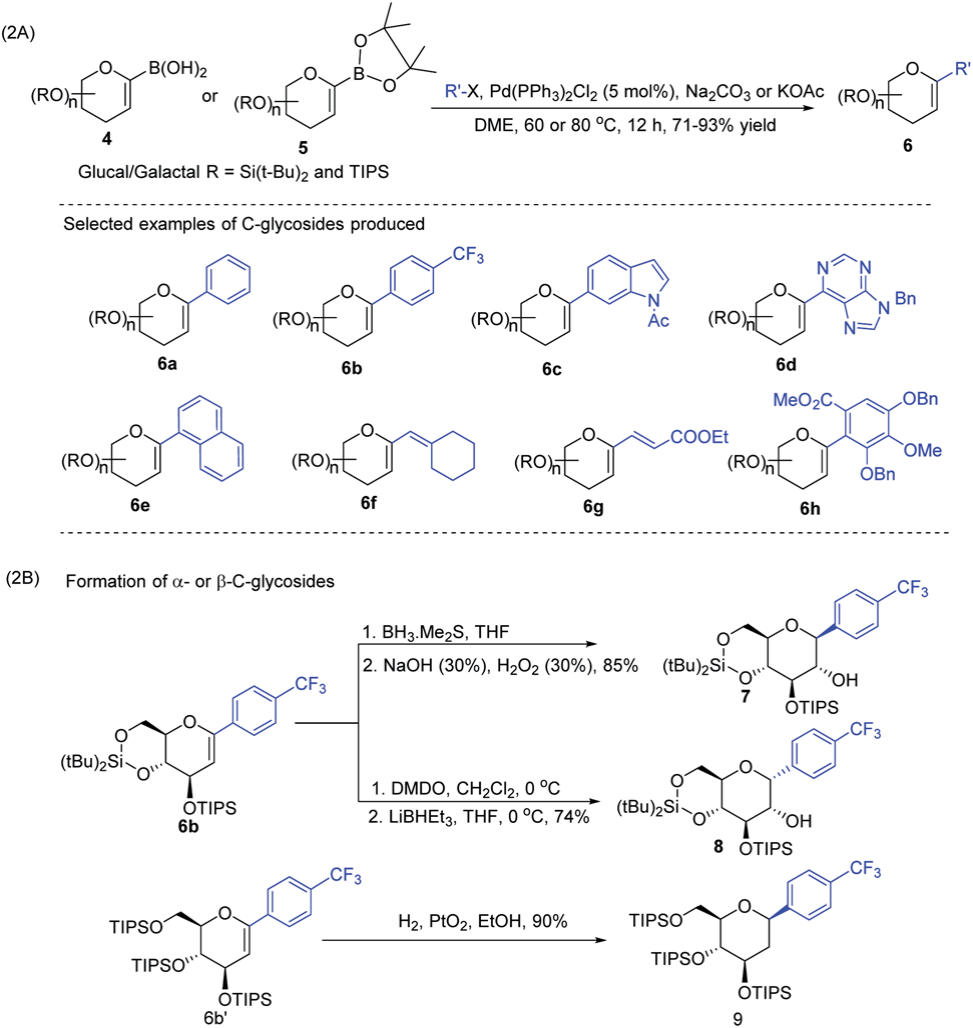
(A) Aryl or heteroaryl or alkenyl-*C*-glycals using Suzuki–Miyaura cross-coupling and (B) formation of the corresponding α- & β-*C*-glycosides.

J. S. Potuzak and D. S. Tan reported the synthesis of alkyl-*C*-glycosides (2) from *C*1-iodoglycal (1) and alkenes using the *B*-alkyl Suzuki–Miyaura cross-coupling approach. A series of alkl-*C*-glycosides (2) was produced by this method and some selected examples 2a–d are shown in Scheme 1A.^27^ Additionally, the authors demonstrated that the palladium-mediated reduction of *C*1-iodoglycals (a side reaction) can be eliminated by pre-incubation of aq. NaOH with alkylborane prior to the addition of the mixture to the *C*1-iodoglycal (1) and palladium catalyst. This protocol was further extended to the synthesis of acyl-*C*-glycosides (3) when the reaction mixture was flushed with carbon monoxide (CO) as depicted in (Scheme 1B). This approach overcomes major the limitations associated with the direct alkylation or acylation of glycals. Pd-catalyzed Suzuki–Miyaura cross-coupling was also exploited by Kamil Parkan *et al.* to synthesize 1-aryl or heteroaryl or alkenyl-*C*-glycals in 87–93% yields using glucal or galactal boronates 4 or 5 as depicted in (Scheme 2A). The 1-aryl or heteroaryl or alkenyl-*C*-glycals (6) so produced can further be converted into α- or β-*C*-glycosides (7–9) in very good yields in a diastereoselective manner by the further functionalization of the double bond (Scheme 2B).^28^

#### Applications in the synthesis of natural products

**Scheme 3:**
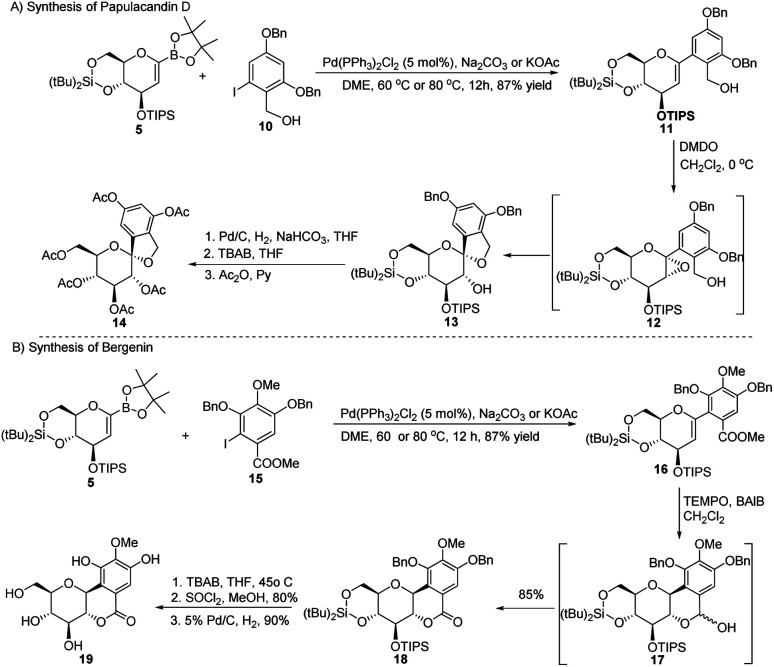
Synthesis of natural products using Suzuki–Miyaura coupling: (A) the papulacandin D glycopyranoside moiety and (B) bergenin.

The synthetic potential of this methodology has been demonstrated in the synthesis of two natural products, namely, papulacandin D glucopyranoside (14) and bergenin (19). The Pd-catalyzed Suzuki–Miyaura cross-coupling between the sugar boronate (5) and iodobenzene derivative (10) to produce glycoside 11 was followed by epoxidation to form the unstable epoxide 12, which was readily converted to 13. After de-protection/protection steps, compound 13 was converted into papulacandin D glucopyranoside (14) in 64% overall yield (Scheme 3A).

The authors explored the Suzuki–Miyaura cross-coupling methodology as a key step in the total synthesis of bergenin (19) starting from the sugar boronate (5) and iodobenzene derivative (15) in 40% overall yield (Scheme 3B).

### 2.2. *C*-Glycosides *via* microwave-assisted palladium-catalyzed cross-coupling

**Scheme 4:**
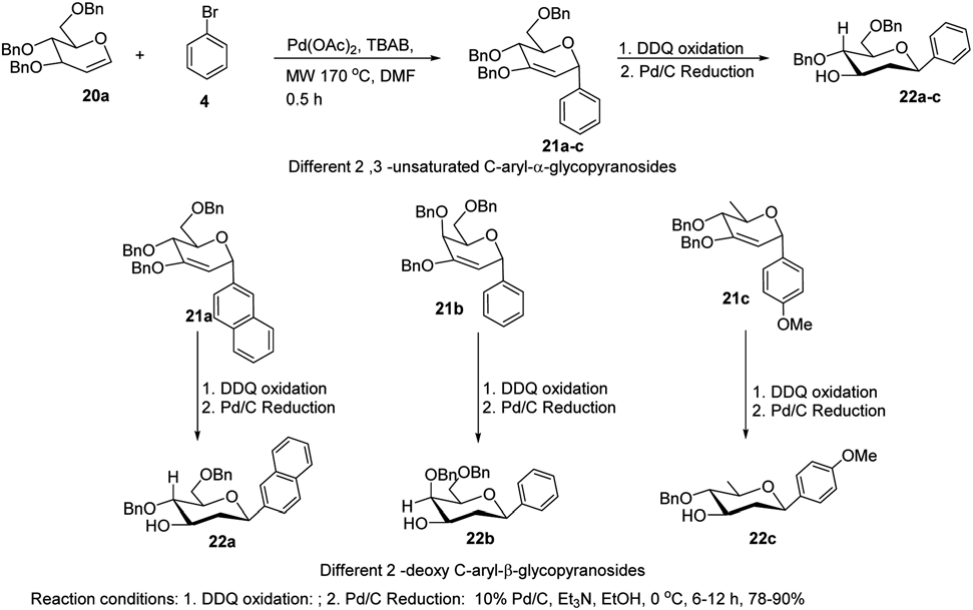
Microwave-assisted Pd(ii)-catalyzed cross-coupling.

The *C-*glycosides are structurally unique carbohydrates with an aromatic moiety directly bonded to the anomeric carbon.^29^ M. Lei *et al.* developed a microwave-assisted Pd(ii)-catalyzed cross-coupling reaction of glycal (20a) with different aryl bromides for the formation of structurally diverse 2,3-unsaturated *C*-aryl-α-glycopyranosides (21a–c) (Scheme 4).^30^ This is a rapid, high-yielding and stereospecific method that offers a more practical approach when compared to previously reported procedures. M. Lei *et al.* further elaborated that the *C*-glycosides so obtained can further be converted into 2-deoxy-*C*-aryl-β-glycopyranosides (22a–c) *via* DDQ-mediated oxidation followed by stereoselective reduction.

### 2.3. *C*-Glycosides *via* palladium-catalyzed organosilanolate-based cross-coupling

**Scheme 5:**
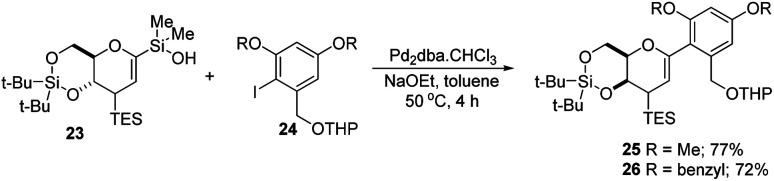
Pd-catalyzed, organosilanolate-based, cross-coupling.

S. E. Denmark *et al.* developed a palladium-catalyzed silicon-based cross-coupling reaction for *C-*arylglycosylation (Scheme 5), wherein they demonstrated the synthetic power of fluoride-free activation for a variety of silanol-containing reagents. This methodology involves the reaction of glucal silanol 23 with aryl iodide 24 to produce *C*-arylglycosides (25–26) in very good yields (77% if R is methyl and 72% if R is benzyl) as depicted in (Scheme 5).^31^

**Scheme 6:**
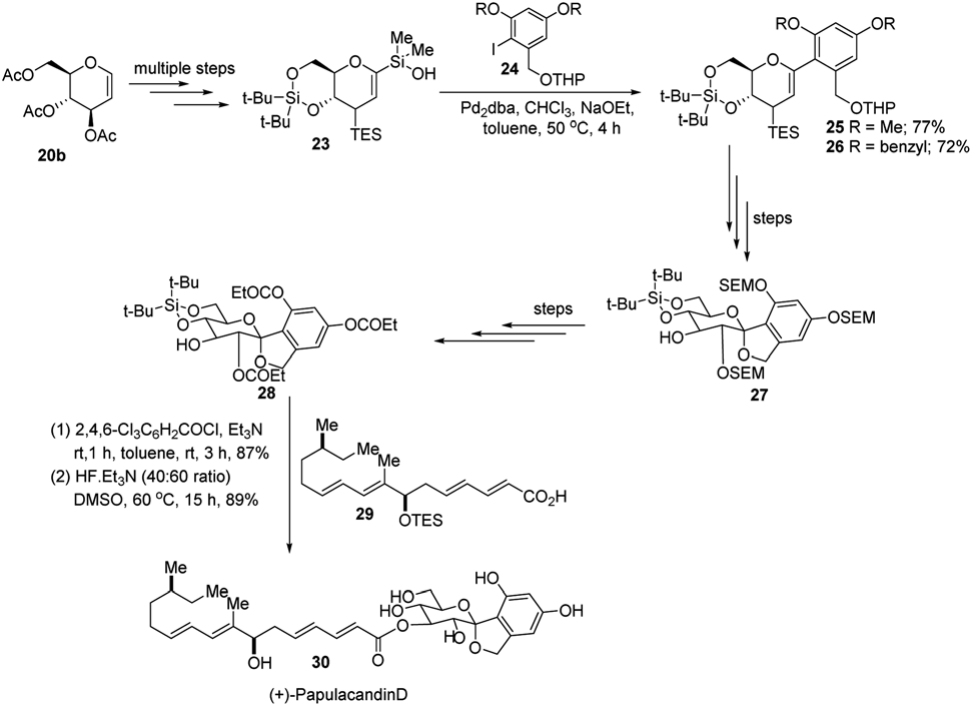
Synthesis of (+)-papulacandin D *via* Pd-catalyzed organosilanolate-based coupling.

S. E. Denmark *et al.* further demonstrated the application of this *C-*arylglycosylation reaction in the total synthesis of (+)-papulacandin D (30), an antifungal agent isolated from the deuteromycetous fungus *Papularia sphaerosperma*.^31^ They accomplished the total synthesis of (+)-papulacandin D (30) in 31 steps with an overall 9.2% yield starting from tri-*O*-acetyl-d-glucal (20b). The retrosynthetic strategy involves the breaking of the molecule into two fragments: (i) *C*-spirocyclic arylglycoside and (ii) the polyunsaturated fatty acid side-chain, obtained from geraniol (29) through several steps. The application of the Pd-catalyzed organosilanolate-based cross-coupling reaction, developed and optimized by S. E. Denmark's group, produced 25 or 26, which after several steps produced 27. The compound 27 was then converted into 28, which on reaction with the compound derived from geraniol (29) produced the (+)-papulacandin D (30) as depicted in (Scheme 6).^32^

### 2.4. *C*-Glycosides *via* the Suzuki coupling-hydroboration-oxidation protocol

**Scheme 7:**
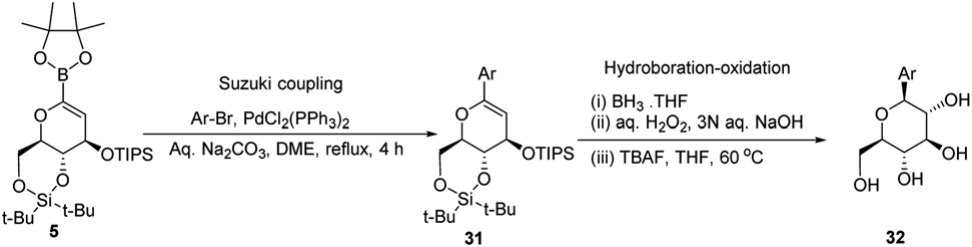
The Suzuki coupling-hydroboration-oxidation protocol.

A novel aryl-β-*C*-glucosidation method was developed by S. Sakamaki's group. This method is a combination of Pd-catalyzed coupling and hydroboration-oxidation. The glucal boronate (5) was subjected to Suzuki coupling to produce 31, followed by hydroboration-oxidation to generate aryl-β-*C*-glucoside 32 (shown in Scheme 7). Glucal boronate (5) is a non-toxic, crystalline solid and can be stored under room temperature conditions for many months, thus, making this protocol advantageous over other methods.^33^

**Scheme 8:**
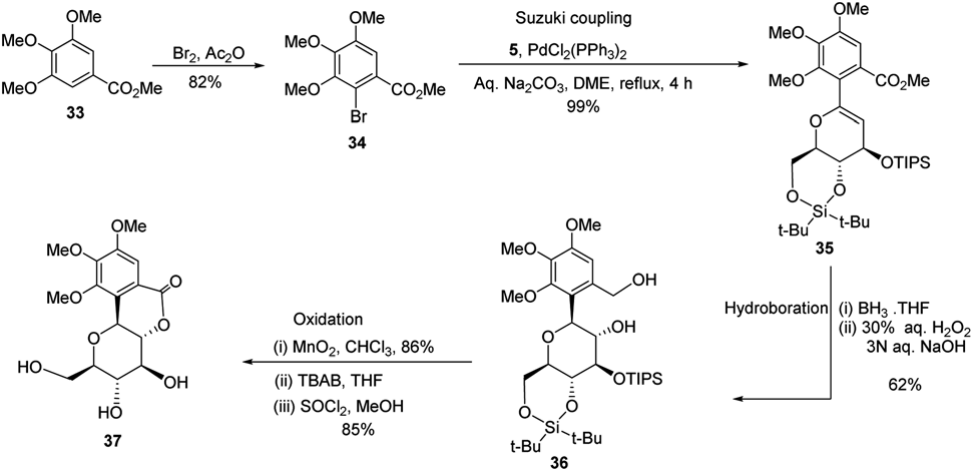
Synthesis of tri-*O*-methylnorbergenin *via* the Suzuki coupling-hydroboration-oxidation.

S. Sakamaki's group further demonstrated the application of this protocol in the synthesis of tri-*O*-methylnorbergenin, an anti-HIV natural product isolated from *Ardisia japonica*.^34^ The ester 33 was brominated to form aryl bromide 34, which was subjected to Suzuki coupling to produce glycoside 35. Glycoside 35 underwent hydroboration to form 36, followed by oxidation to give the targeted natural product 37. Starting from glucal boronate (5), the synthesis of tri-*O*-methylnorbergenin (37) was completed with 47% overall yield (Scheme 8), which is better than the previously reported yields (33%).^35^

### 2.5. *C*-Glycosides *via* Pd-catalyzed coupling of glycals with arene diazonium salts

**Scheme 9:**
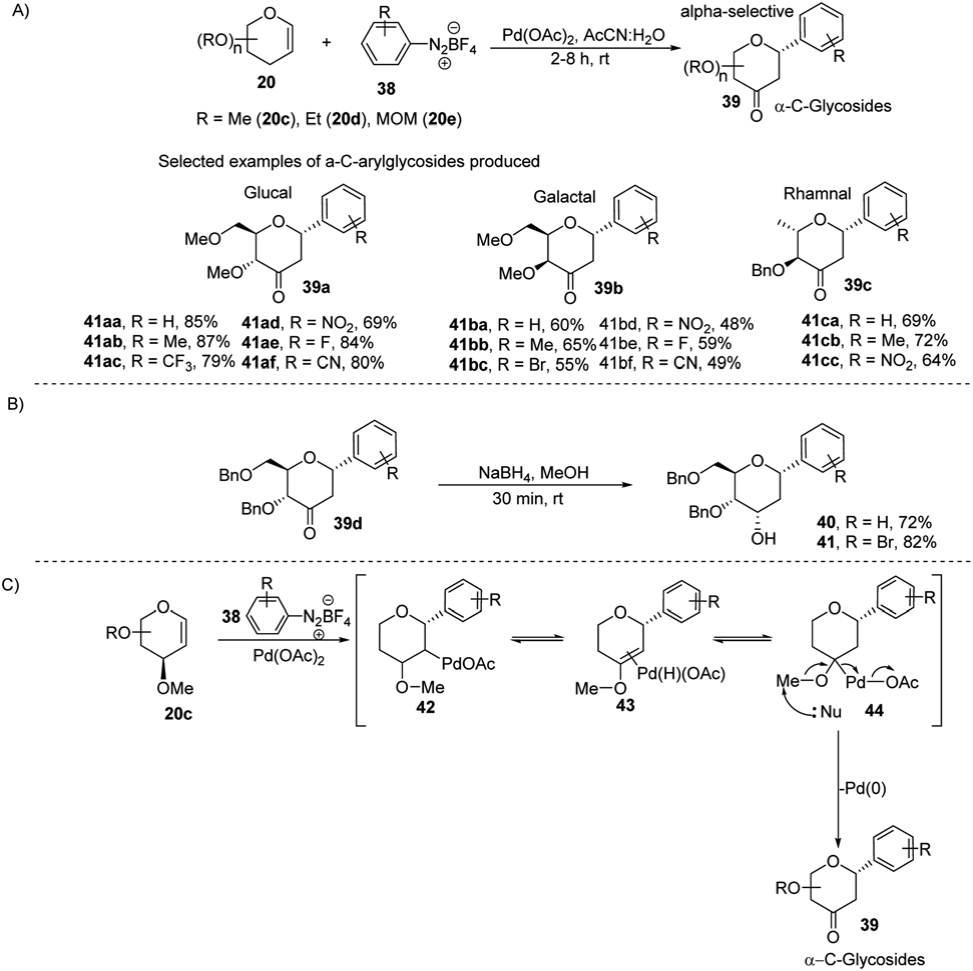
(A) *C*-Glycosides *via* the Pd-catalyzed coupling of glycals with arenediazonium salts (B) reduction of keto group (C) plausible mechanism.

The electrophilic character of diazonium salts might allow them to be used in glycosyl coupling reactions in the absence of ligand or base under milder reaction conditions and generate aryl-*C*-glycosides.^36,37^ Due to their continuous interest in glycosylation reactions, J. Kandasamy *et al.* reported a method wherein they made use of glycals (20) and aryldiazonium salts (38) for the synthesis 2,3-deoxy-3-keto-α-aryl-*C*-glycosides (39) in the presence of Pd-catalyst (Scheme 9A).^38^ The reaction conditions are compatible with a wide range of substrates (glycals) like d-glucal, d-galactal, l-rhamanal, d-xylal and d-ribal. The room temperature conditions and simple operation make this process very attractive in synthetic chemistry. The synthesized keto forms of the *C*-glycosides (39a–d) can serve as the starting materials for the synthesis of 2-deoxy *C*-aryl glycosides (40–41) using NaBH_4_ reduction as demonstrated in (Scheme 9B).

The authors proposed a plausible mechanism for the palladium-catalyzed *C*-arylation of glycal (20c). It involves the following four steps: (i) oxidative addition, (ii) β-hydride elimination,^39^ (iii) palladium-hydride reinsertion, and (iv) reductive elimination. The various intermediates (42–44) involved in the mechanism are shown in (Scheme 9C).

### 2.6. *C*-Glycosides using Heck coupling

**Scheme 10:**
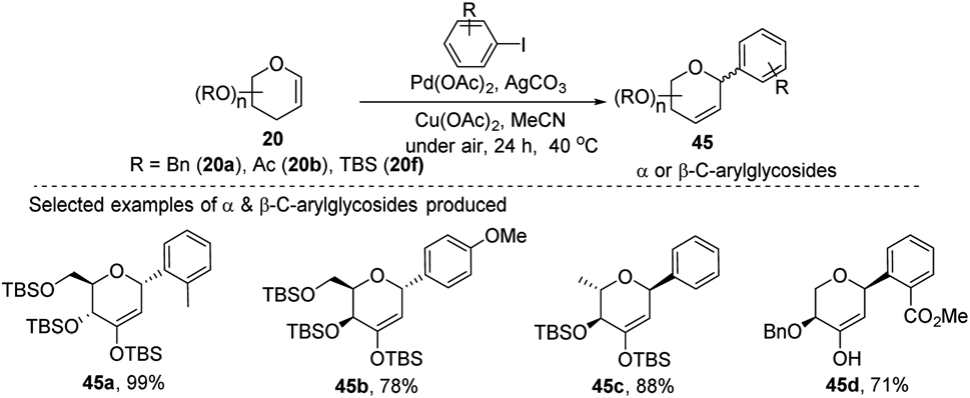
α and β-*C-*diaryl-glycosides *via* simple Heck coupling.

A simple Heck coupling approach was reported by Xin-Shan Ye's group, which involves the coupling of glycals (20c–e) with various aryl iodides to selectively synthesize α- or β-aryl-2-deoxy-*C*-arylglycosides (45a–d, only selected examples shown in Scheme 10). This protocol is characterized by the following salient features: (i) no use of air-sensitive phosphorous ligands, (ii) easy operation under air, (iii) regio- and stereo-selective as only a single anomer is produced, (iv) the Heck reaction proceeds through *syn* β-hydride elimination, and (v) no ring-opening products or carbon-Ferrier-type products. Since the aryl halides are more easily available than aryl boronic acids, this procedure may find wide applications in the synthesis of naturally occurring 2-deoxy-aryl *C*-glycosides and pyranoid *C*-nucleosides having medicinal importance.^40^

**Scheme 11:**
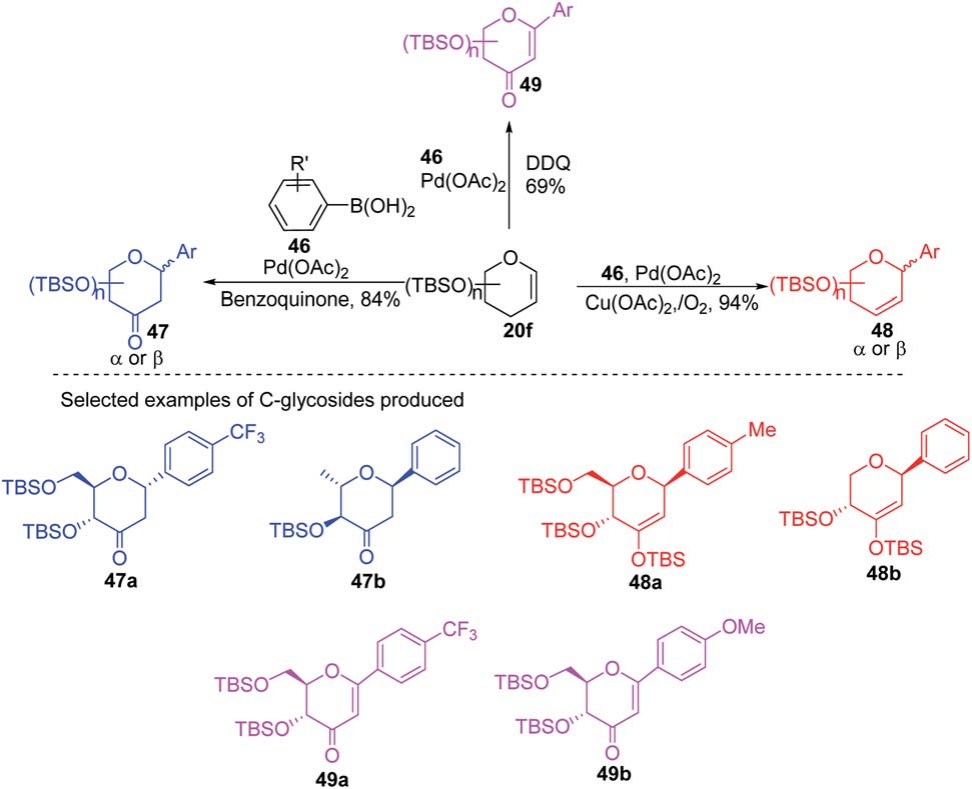
Synthesis of *C-*glycosides *via* oxidant-controlled Heck coupling.

Due to their continuous interest in *C-*glycosidations Xin-Shan Ye's group further extended their studies and devised an oxidant-controlled Heck-type *C*-glycosylation protocol wherein they reacted different TBS-protected glycals (20f) with various arylboronic acids (46) in the presence of a catalytic amount of Pd(OAc)_2_ and an oxidant (Scheme 11). As the name suggests, it is an oxidant-controlled coupling and the product outcome could be changed by changing the oxidant. Thus, with benzoquinone (BQ) as the oxidant, ketone-type *C*-glycosides (47a–b, only selected examples are shown) are produced, while Cu(OAc)_2_/O_2_ as the oxidant produces enol ether type *C*-glycosides (48a–b, only selected examples are shown), and with 2,3-dichloro-5,6-dicyano-1,4-benzoquinone (DDQ) as the oxidant, the enone type *C*-glycosides are produced (49a–b, only selected examples are shown). Apart from other advantages associated with arylboronic acids, this oxidant-controlled Heck-type coupling provides a simple, mild, and stereoselective synthesis of aryl 2-deoxy-*C*-glycosides. This protocol may find wide significance in the synthesis of many biologically relevant *C*-glycosides.^41^

**Scheme 12:**
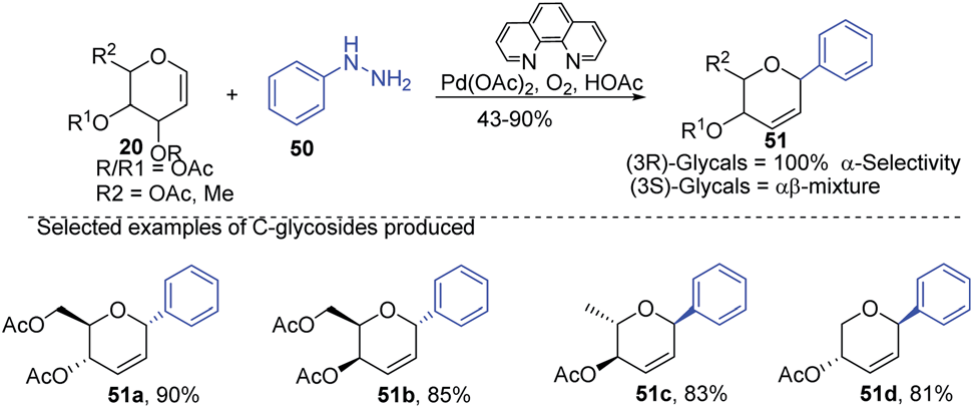
Synthesis of *C-*glycosides using Heck coupling *via* C–N bond cleavage.

Liu *et al.* proposed that when glycals are protected by good leaving groups (like acetyl), the Heck reaction involves β-heteroatom elimination to produce 2,3-deoxy-*C*-glycosides. Therefore, Liu's group devised a method for the synthesis of 2,3-deoxy-*C*-glycosides (51a–d) through the Heck coupling of glycals (20) with aryl hydrazines (50) as shown in (Scheme 12). This procedure involves the C–N bond cleavage of aryl hydrazines. The reaction is highly stereoselective as (3*R*)-glycals produce 100% α-*C*-glycosides, while (3*S*)-glycals produce α, β mixtures.^42^

**Scheme 13:**
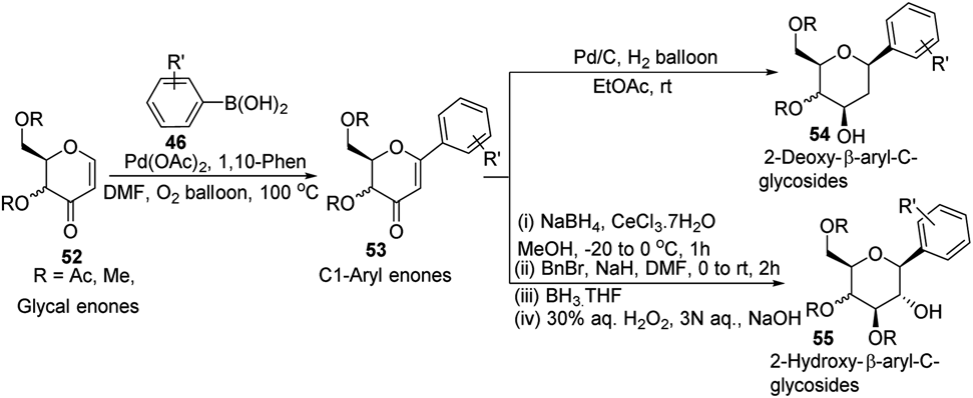
Synthesis of 2-deoxy- & 2-hydroxy-β-aryl-*C*-glycosides *via C*-1 aryl enones.

In another Heck coupling, reported by J. Kandasamy's group, they devised a two-step protocol for achieving 2-deoxy-β-aryl-*C*-glycosides (54) and 2-hydroxy-β-aryl-*C*-glycosides (55) from glycal-enones (52). The two steps involved in this protocol include (i) the synthesis of *C*-1 aryl enones (53) *via* the regioselective Heck coupling from glycal enones (52) and (ii) synthesis of *C-*glycosides 54 and 55 through the stereoselective reduction of *C*-1 aryl enones (53) as depicted in Scheme 13.^43^

### 2.7. *C*-Glycosides *via* the domino Heck–Suzuki reaction

**Scheme 14:**
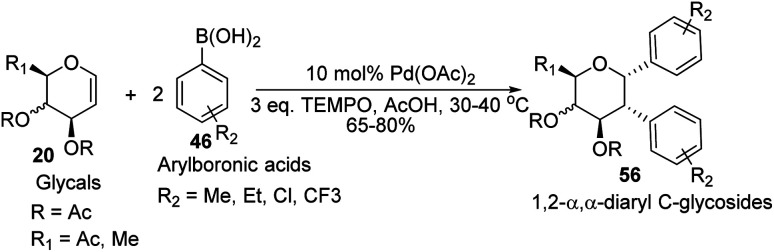
Synthesis of 1,2-α,α-diaryl-*C*-glycosides *via* the domino Heck–Suzuki reaction.

D. Mukherjee's group developed a type of domino Heck–Suzuki diarylation protocol that produced α-*C*-aryl glycosides (56) with *C*1–*C*2 (α,α) selectivity from easily available glycals (20) (Scheme 14).^44^ This palladium-catalyzed protocol is regio- as well as diastereoselective and worked well with a variety of arylboronic acids (46). In this method, *syn*- and *anti*-elimination (side reaction) is blocked by TEMPO, an oxidizing agent.

**Fig. 3:**
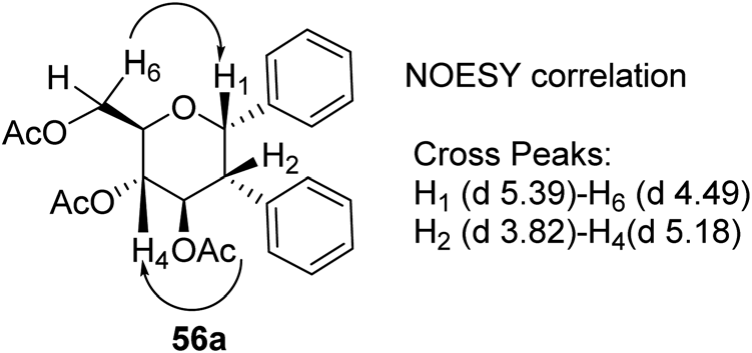
NOESY correlation diagram of diaryl glycosides.

The stereochemistry of aryl moieties as *cis* (α–α) at *C*(1)/*C*(2) was confirmed by the NOESY spectrum of diaryl derivative of tri-*O*-acetyl-d-glucal (56a), where correlations between *H*1/*H*6 and *H*2/*H*4 revealed that *H*-1, *H*-2, *H*-4, and *H*-6 are co-facial. Similarly, the stereochemistry of 3,4-di-*O*-acetyl-l-rhamnal derivative is *cis* (β–β) at *C*(1)/*C*(2) (Fig. 3).

**Scheme 15:**
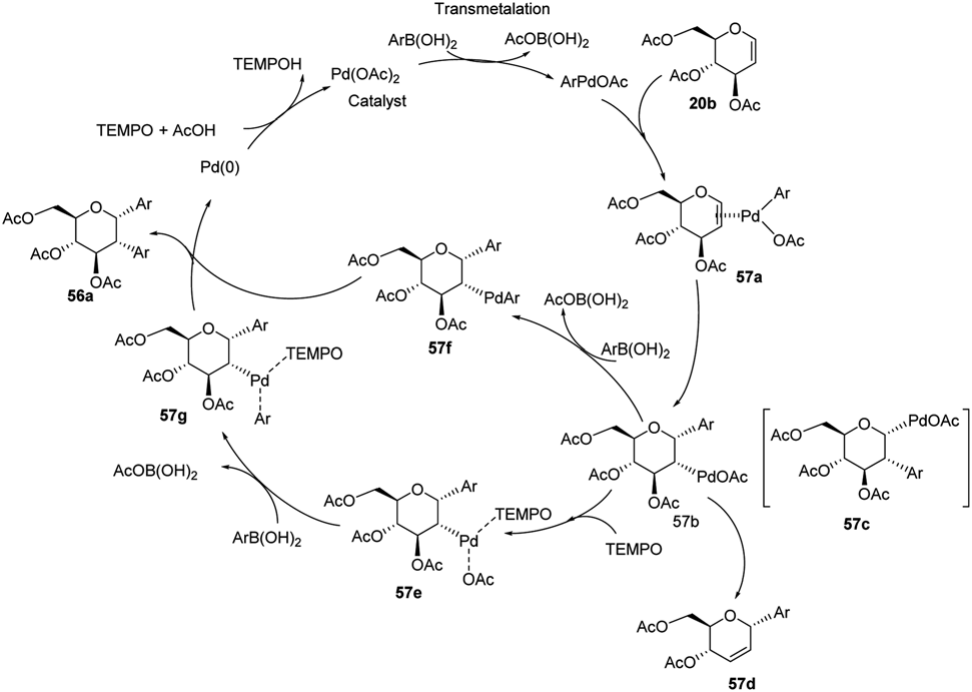
A plausible mechanism for the Domino Heck–Suzuki reaction.

From the above observations, the authors proposed a plausible mechanism for this reaction. D. Mukherjee *et al.* carried out quantum chemical analysis to establish the mechanism of this Pd-catalyzed protocol where Pd(ii)/Pd(0) species are involved. The radical nature of TEMPO allows it to play a significant role in the formation of diaryl glycosides. The detailed mechanism showing various species (57a–g) is given in (Scheme 15).^44^

### 2.8. *C*-Glycosides *via* the Pd-catalyzed C–H functionalization strategy

**Scheme 16:**
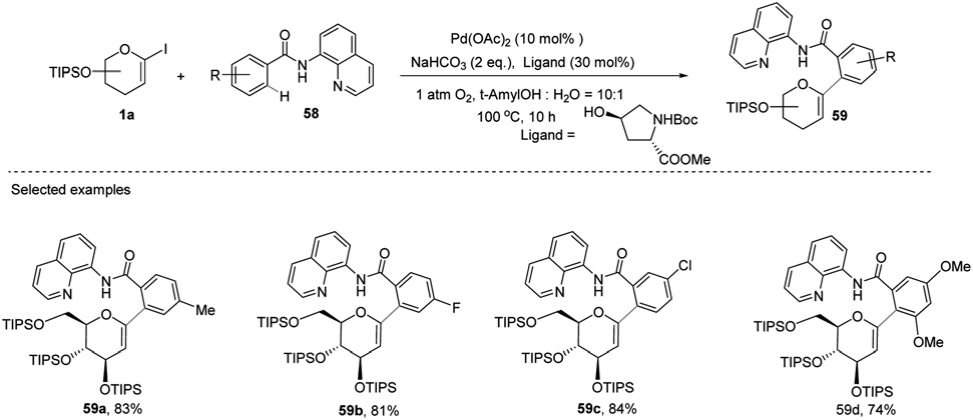
Synthesis of *C-*glycosides *via* ligand-controlled monoselective *C*-aryl glycosylation.

Transition-metal-catalyzed, C–H functionalization has emerged as a powerful method for the construction of organic molecules in the last two decades.^45^ In this endeavor, Xin-Shan Ye's group reported a monoselective synthesis of aryl-*C*-Δ^1,2^-glycosides (59a–d) *via* the Pd-catalyzed *ortho*-C–H activation of *N*-quinolyl benzamides (58) from 1-iodoglycals (1a) as depicted in (Scheme 16).^46^ The use of an amino acid derivative as a ligand is the key factor for improving the mono-selectivity and yield.

**Scheme 17:**
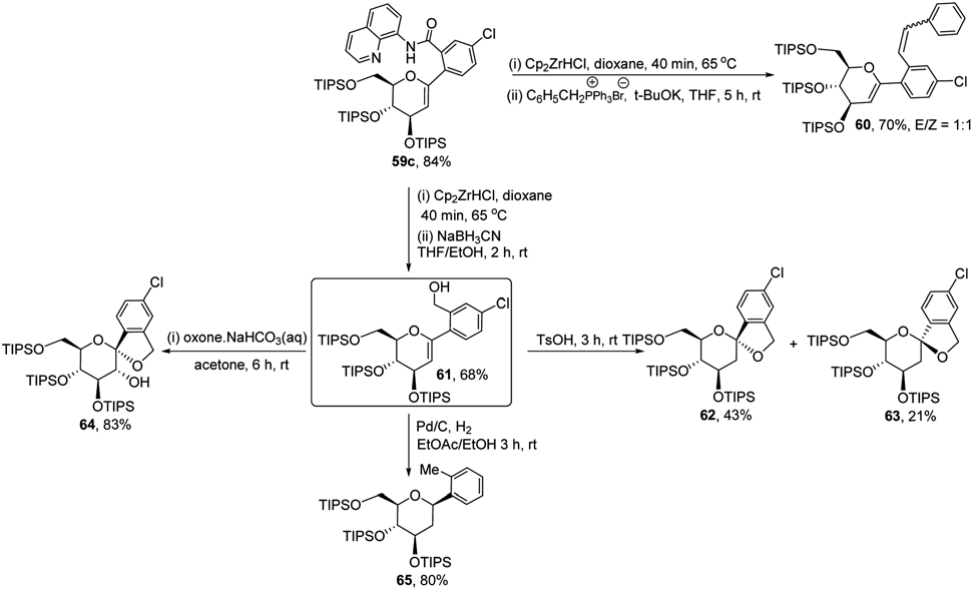
Applications of ligand-controlled monoselective *C*-arylglycosylation.

The authors demonstrated the utility of this protocol by synthesizing the basic scaffolds (60–65, Scheme 17) of some bioactive natural products and drugs such as SGLT2 inhibitor, papulacandin, dapagliflozin, stilbene *C*-glucoside, *etc.*

**Scheme 18:**
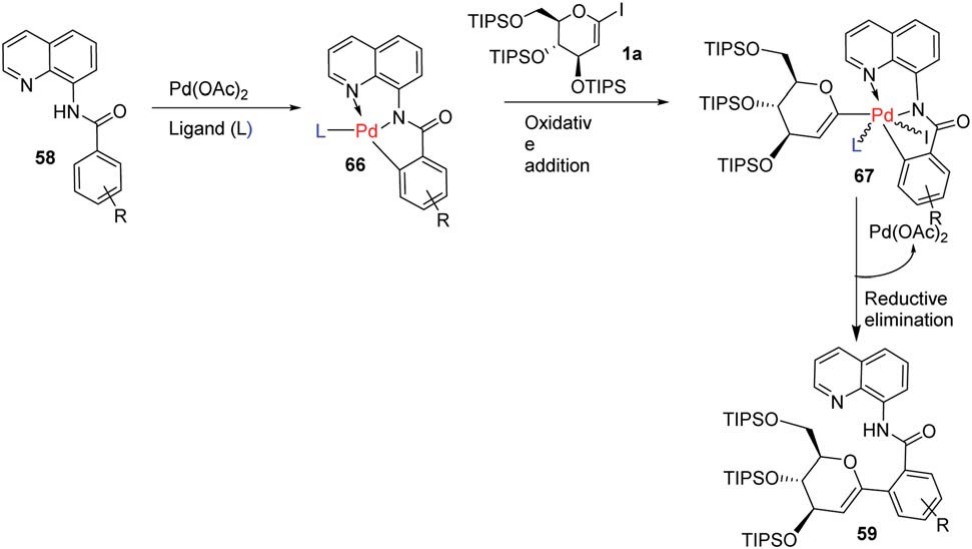
A plausible mechanism of ligand-controlled monoselective *C*-aryl glycosylation.

The mechanism of this reaction involves the coordination of Pd with *N*-quinolyl benzamide (58) and amino acid ligand (L) to generate a palladacycle intermediate (66) through insertion between the C–H bond. This is followed by the rate-determining oxidative-addition of 1-iodoglycal (1a) to form 67, which undergoes reductive elimination to produce the targeted product 59 (Scheme 18). The authors proposed that the amino acid ligand decreases the rate of the first oxidative addition by coordinating to the palladacycle intermediate. This makes the second oxidative-addition more difficult, leading to the high monoselectivity and good yield.^46^

After the successful development of the Pd-catalyzed *ortho*-C–H activation of *N*-quinolyl benzamides for *C-*glycosylation, Xin-Shan Ye's group devised another method for the synthesis of heteroaryl-*C*-glycosides *via* Pd-catalyzed C–H activation of 5-membered *N*-heterocycles.

**Scheme 19:**
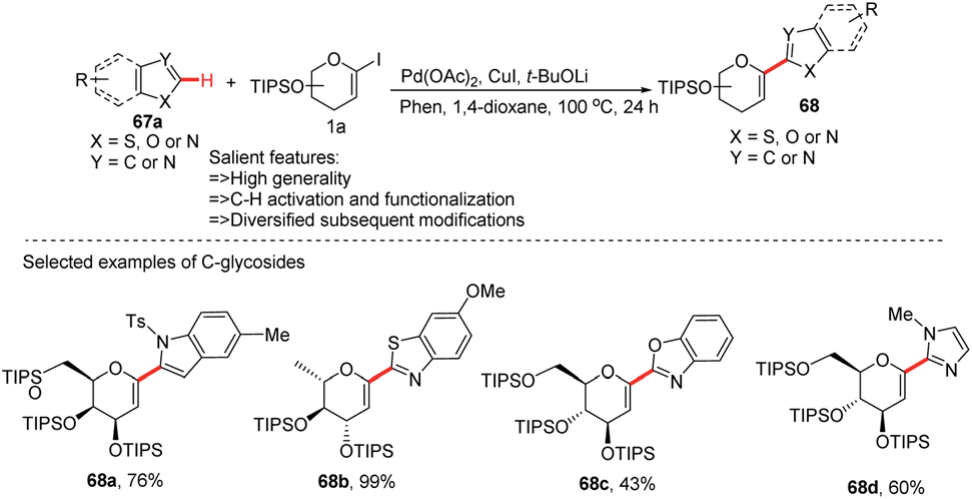
*C*-Heteroaryl glycosides *via* Pd/Cu co-catalyzed C–H functionalization.

In this strategy, they employed the Pd(OAc)_2_/CuI co-catalyzed cross-coupling of 5-membered *N*-heterocycles (67a) with 1-iodoglycals (1a) by activating the C–H bond and produced a series of heteroaryl-*C*-Δ^1,2^-glycosides (68a–d) in 43–99% yield (Scheme 19).^47^ These contain indoles, thiazoles, benzothiazoles, imidazoles, benzimidazoles, and benzoxazoles as an aglycones. This protocol may find wide applications in the preparation of biologically important compounds containing heterocycles.

### 2.9. *C*-Glycosides *via* Pd-catalyzed decarboxylative allylation/Wittig reaction

**Scheme 20:**
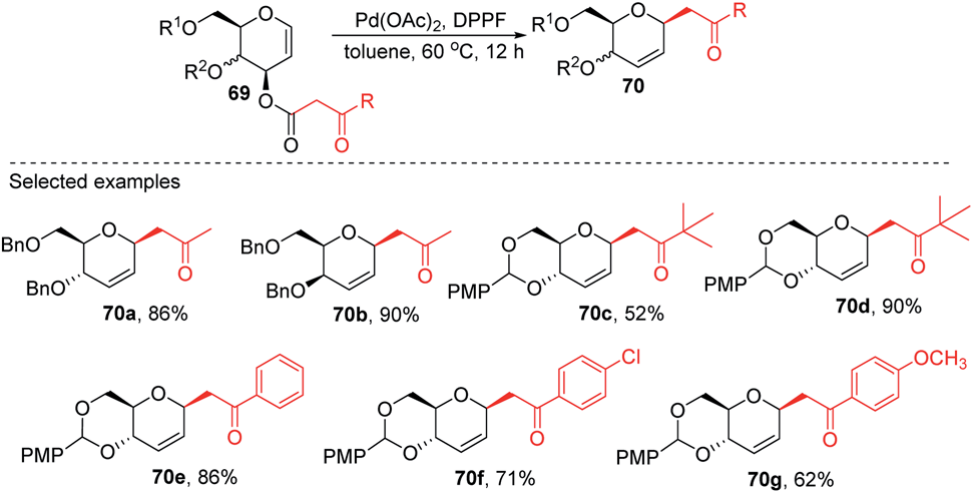
Pd-catalyzed decarboxylative *C-*glycosylation.

Inspired by the works of Tunge, Trost, and Stoltz on catalytic decarboxylative allylation,^48–50^ Liu's group proposed that *C*-glycosylation with high stereoselectivity could be achieved by the Pd-catalyzed decarboxylation of the *C*-3 ester of glycal through the Pd π–allyl intermediate. They successfully developed a Pd-catalyzed decarboxylative *C*-glycosylation of glycal derivatives (69) through a tandem sequence of rearrangement and decarboxylation on the sugar moiety to produce 2,3-deoxy-*C*-glycosides (70a–g) (Scheme 20).^51^ The salient features of this method are its versatility, flexibility, extensive substrate scope, high yields, exclusive regioselectivity, diasteroselectivity and gram-scale applicability. The natural glycoconjugates contain *C-*glycosides, therefore, this method has the potential to access natural products.

**Scheme 21:**
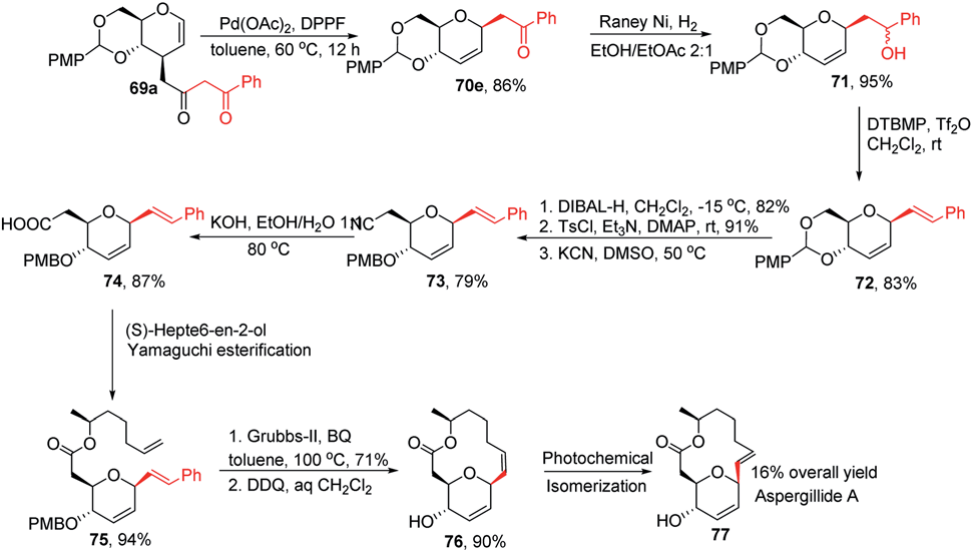
Synthesis of aspergillide A using Pd-catalyzed decarboxylative *C-*glycosylation.

This was demonstrated by the authors by employing this strategy for the synthesis of the natural product aspergillide A in 16% overall yield involving various intermediates (70e–77) starting from the glucal derivative (69a) (Scheme 21).^51^ The *Z*-isomer (76) formed in the second-to-last step was photochemically isomerized to the *E*-isomer, *i.e.*, aspergillide A (77) using the literature method.^52^

**Scheme 22:**
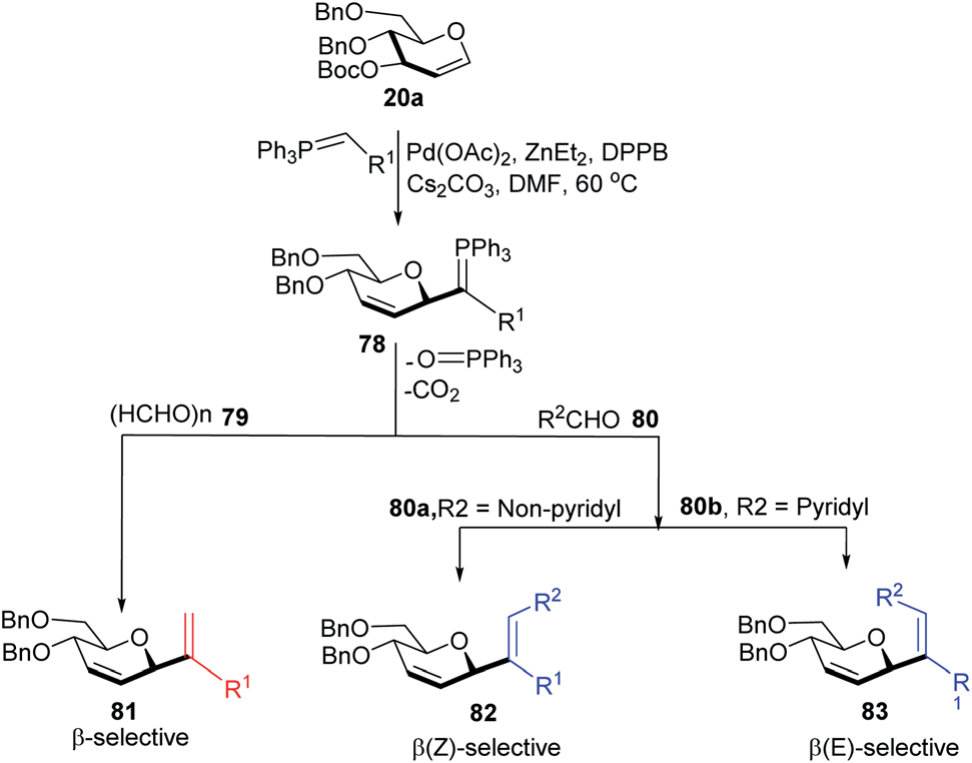
*C*-Vinyl glycosides *via* Pd-catalyzed decarboxylative allylation/Wittig reaction.

Xue-Wei Liu's group further extended their studies on Pd-catalyzed decarboxylative *C-*glycosylations and devised a Pd-catalyzed Tsuji–Trost-type decarboxylative allylation/Wittig protocol to generate diastereoselective *C*-vinyl glycosides (81–83) from glucal (20a) in controlled diastereoselectivity (Scheme 22). They demonstrated that the successive generation of two stereo-centers led to diastereoselectivity and it depends on the coordinating ability of the aldehydes. With formaldehyde (79), β-selective *C*-vinyl glycosides are produced (81). The non-pyridyl aldehydes (80a) generated β,(*Z*)-selective *C*-vinyl glycosides (82) while the pyridyl aldehydes (80b) generated β,(*E*)-selective *C*-vinyl glycosides (83) as depicted in Scheme 22.^53^ These *C*-vinyl glycosides could be useful synthetic precursors for the synthesis of natural products and pharmaceuticals if subjected to downstream functionalization.

**Scheme 23:**
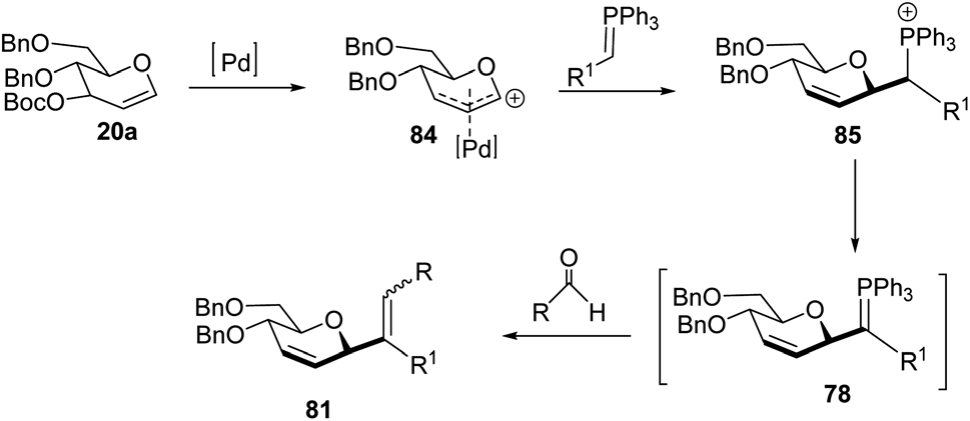
*C*-Vinyl glycosides *via* Pd-catalyzed decarboxylative allylation/Wittig reaction.

The authors proposed two mechanistic pathways based on observed diastereoselectivity: (i) the pseudo-intramolecular mechanism based on Pd–N coordination and (ii) the classic intermolecular mechanism in the absence of coordination. The Tsuji–Trost reaction proceeds *via* an outer sphere pathway that gives rise to β-selectivity due to the soft nature of the *P*-ylide nucleophile (Scheme 23).

**Scheme 24:**
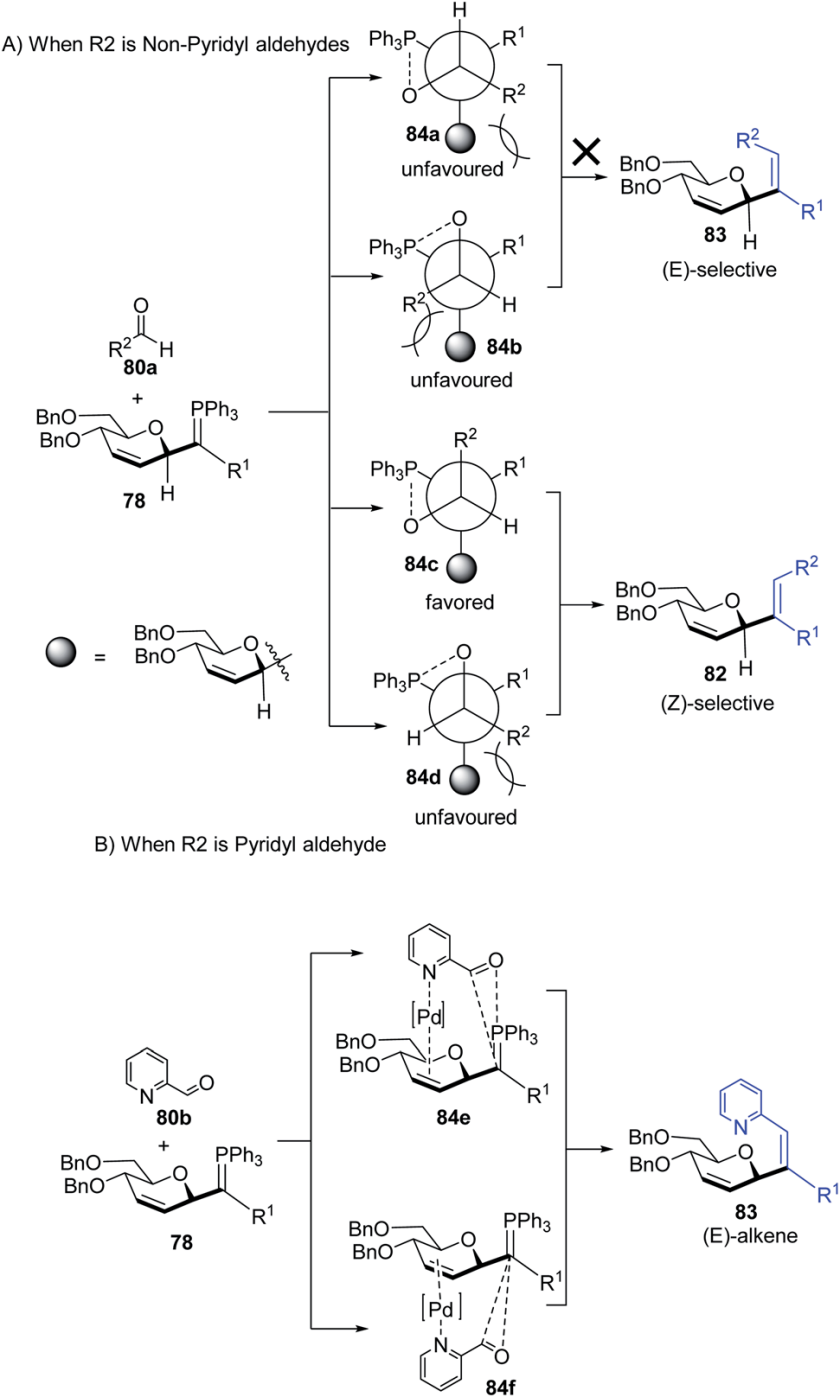
Proposed oxaphosphetane intermediates accounting for (A) (*Z*)- (B) (*E*)-selectivity.

The substituents on the aldehydes determine the olefin selectivity in the case of the Wittig reaction (Scheme 24). In the case of non-pyridyl aldehydes (80a) where R^2^ = phenyl, furyl, and thienyl aldehydes, the classical intermolecular Wittig pathway is followed to give (*Z*)-alkenes (82). The (*Z*)-selectivity is favored due to the preferential generation of oxaphosphetane with the aldehyde substituent anti to the sugar group (Scheme 24A). However, in the case of pyridyl aldehydes (80b), they proposed Pd–N coordination,^54^ which led to (*E*)-selectivity (83). The Wittig reaction proceeds through a pseudo-intramolecular route due to Pd–N coordination, which brings the *P*-ylide and aldehyde into close proximity for [2 + 2] cycloaddition, thus, overcoming the opposing steric factors in the usual Wittig reaction, which usually limits the efficiency of the formation of trisubstituted alkenes (Scheme 24B).

### 2.11. *C*-Glycosides *via* Stille-coupling using diaryliodonium triflates

**Scheme 25:**
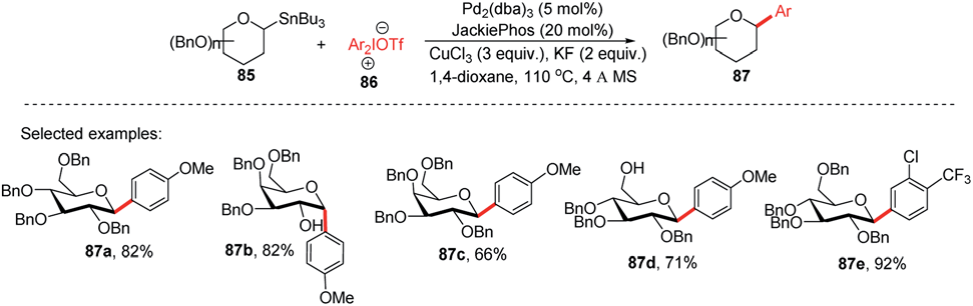
*C*-Glycosides *via* stereoretentive Stille-coupling using diaryliodonium triflates.

Since the *C*-glycosides play a significant role in drug discovery and medicinal chemistry, methods that enable the introduction of the glycosyl group with predictable and high stereochemical outcomes are highly desired.^55,56^ In order to have access to aryl *C*-glycosides of biomedical relevance, M. A. Walczak's group reported an intermolecular glycosyl cross-coupling method that finds applications in drug discovery programs and is well suited for late-stage diversification. This method allows the introduction of glycans in a programmed and predictable fashion. This protocol involves the stereo-retentive *C*-glycosylation of anomeric stannanes (85) with diaryliodonium triflates (86) and allows the retention of anomeric aryl *C*-glycosides of biomedical relevance (87a–e, selected examples shown in this review) as depicted in (Scheme 25).^57^

**Scheme 26:**
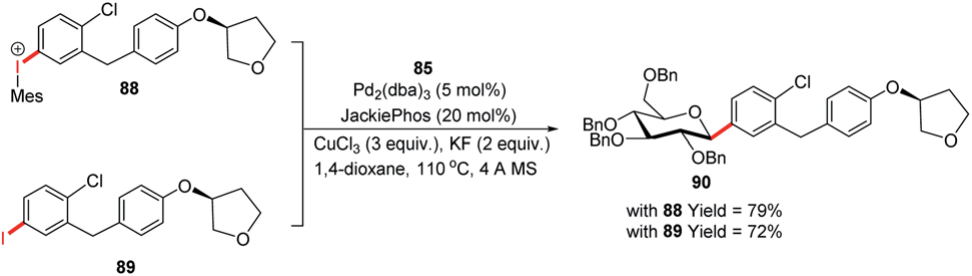
The synthesis of Empagliflozin *via* Stille-coupling using diaryliodonium triflates.

The authors demonstrated the utility of this glycosyl cross-coupling protocol by synthesizing the protected empagliflozin (90), an anti-diabetic drug that is an inhibitor of sodium-glucose co-transporter-2.^58^ They applied the glycosyl cross-coupling protocol to anomeric stannane (85) and mesityl iodonium (88) or iodide (89) and produced 90 as a single anomer in 72–77% yield (Scheme 26).

### 2.12. *C*-Glycosides *via* Stille-coupling using aryl halides

**Scheme 27:**
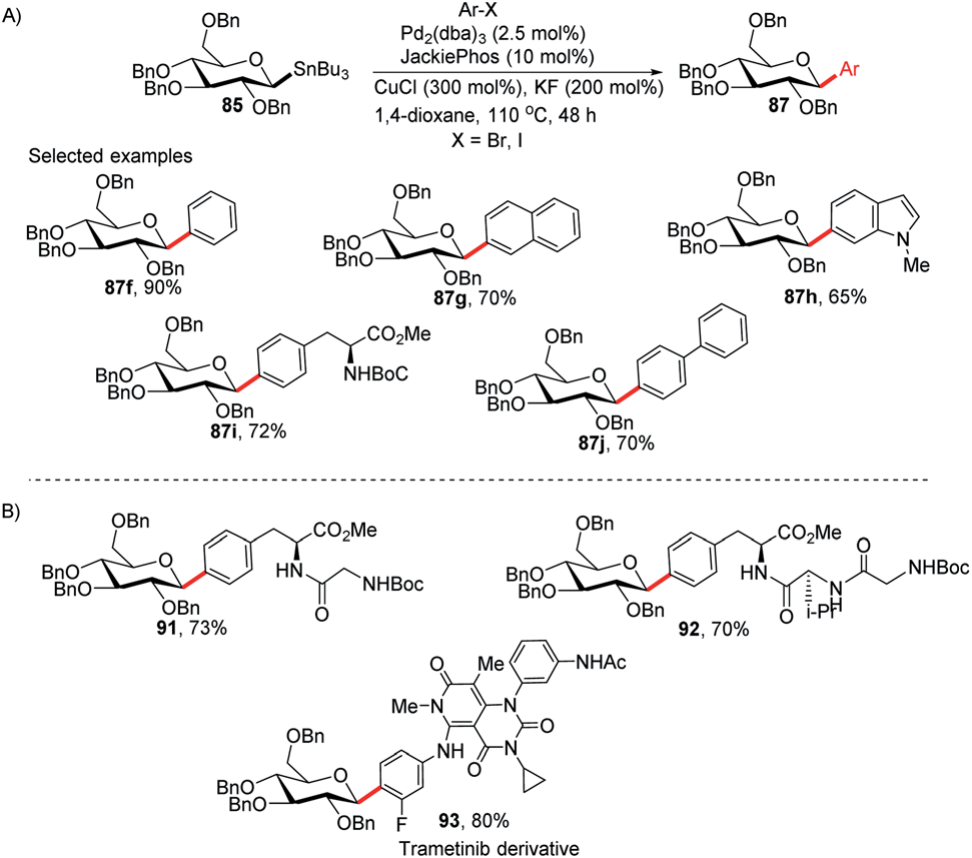
(A) *C*-Glycosides *via* intermolecular Stille-coupling using aryl halides (B) applications of *C*-glycosides.

Walczak's group, for the first time, reported a palladium-catalyzed intermolecular Stille coupling protocol for the synthesis of *C*-glycosides (87f–j) from configurationally stable anomeric stannanes (85) in a cross-coupling reaction with aromatic halides (Ar–X, X = Br, I) (Scheme 27A).^59^ The reaction is stereospecific and a high level of stereo-control was reported. This method is applicable to monosaccharides, oligosaccharides, peptides, heteroaromatics and pharmaceuticals and eliminates many problems associated with previous nucleophilic displacement methods. Furthermore, this method creates new opportunities to incorporate saccharides into small molecules and biologics because of its exceptionally broad substrate scope and high chemoselectivity.^60^ The authors synthesized glycoconjugates 91 and 92 with β-selectivity and converted trametinib, an MEK inhibitor used as an anticancer drug, into *C*-glucoside 93 (Scheme 27B). Thus, this coupling method could be used as a tool for the modification of peptides and small proteins^61^ at specific sites and thus, will open mechanisms to identify new drug candidates with improved pharmacokinetic profiles and stabilities.

#### Applications in the synthesis of natural products and drugs

**Scheme 28:**
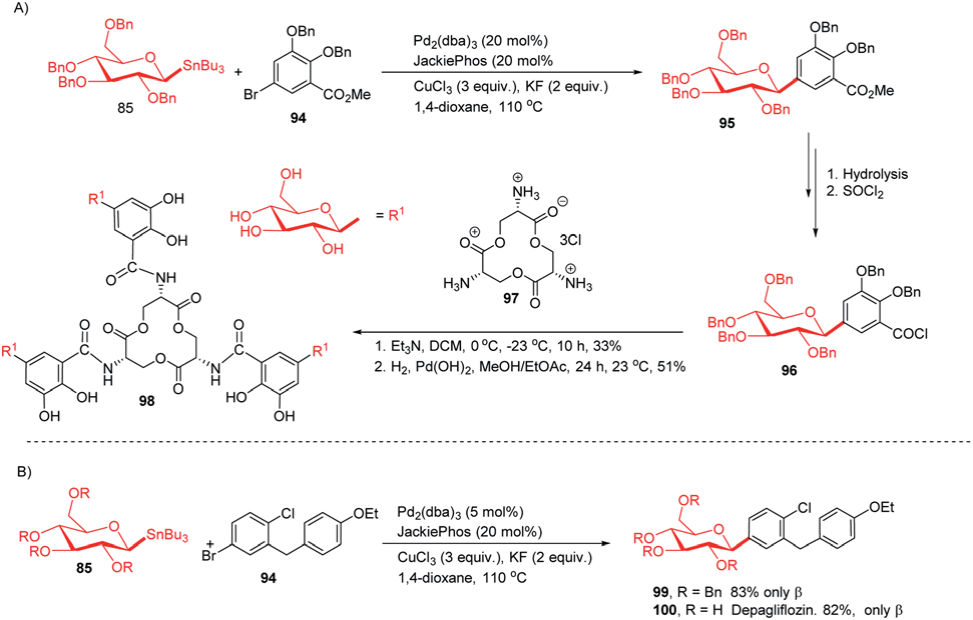
(A)*C*-Glycosides *via* intermolecular Stille-coupling using aryl halides (B) applications of *C*-glycosides.

The applicability of optimized intermolecular Stille coupling was demonstrated by synthesizing the natural product Salmochelin TGE (98)^62,63^ and dapagliflozin (100), an anti-diabetic drug. Compound 98 is produced by Salmonella species^64^ and is a triply glycosylated salmochelin. The total synthesis of TGE involves the coupling of 5-bromobenzoate (94) with β-d-glucose stannane (85) to produce *C*-glycoside (95) in 77% yield with *α* : *β* greater than 1 : 99. After a few manipulations on the ester group, *C*-glycoside (96) was produced, which, on reaction with amine salt (97) followed by removal of the protecting groups, produced TGE (98) in 51% yield (Scheme 28A).^65^ Dapagliflozin (100) is an SGLT2 inhibitor used to treat diabetes mellitus type 2.^66–68^ The authors synthesized dapagliflozins (99 & 100) by applying this Stille-coupling protocol to protected stannane (85) with bromobenzoate (94) in 83% yield (Scheme 28B).^69^

#### Proposed mechanism of Stille coupling

**Scheme 29:**
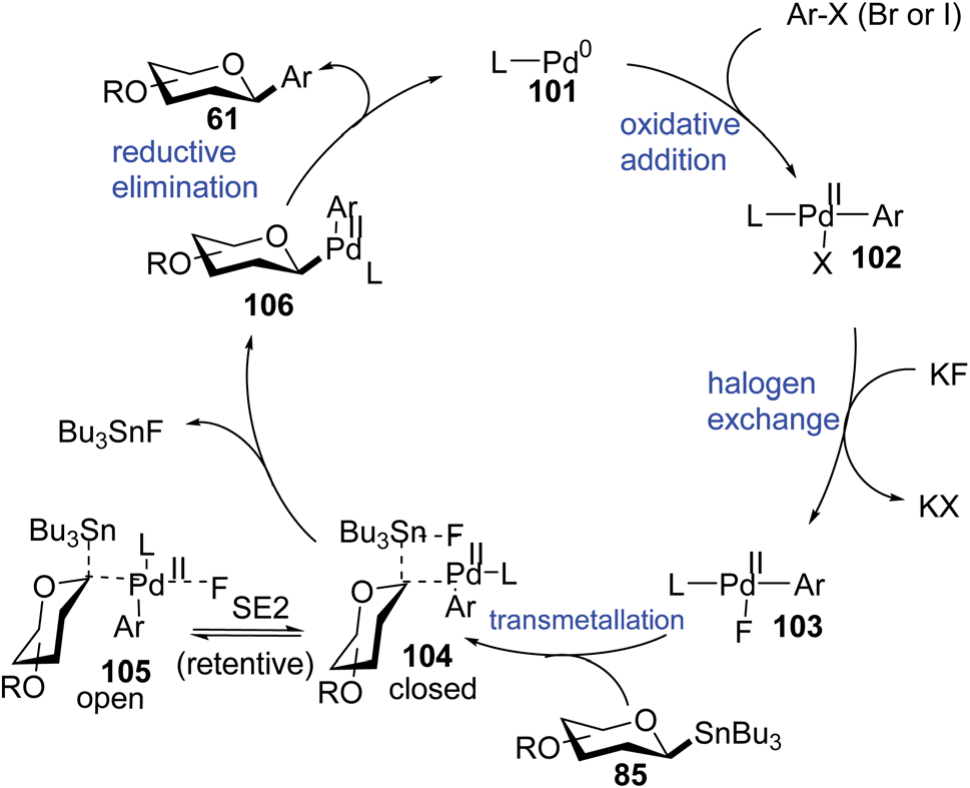
Catalytic cycle of Pd-catalyzed Stille-coupling with anomeric stannanes.

High stereospecificity, broad substrate scope, excellent compatibility with functional groups in Stille-coupling for both anomers prompted the authors to undertake mechanistic studies. Walczak *et al.* proposed a palladacycle mechanism based on density functional theory (DFT) calculations and radical scavenging experiments taking β-d-glucose stannane (85) and bromobenzene (Ar–Br) as model substrates. The complete mechanism involving various intermediate species (101–106) is demonstrated in Scheme 29.^69^

### 2.13. *C*-Glycosides *via* Stille-type coupling using C(sp^2^)- and C(sp^3^)-thio(or seleno)esters

**Scheme 30:**
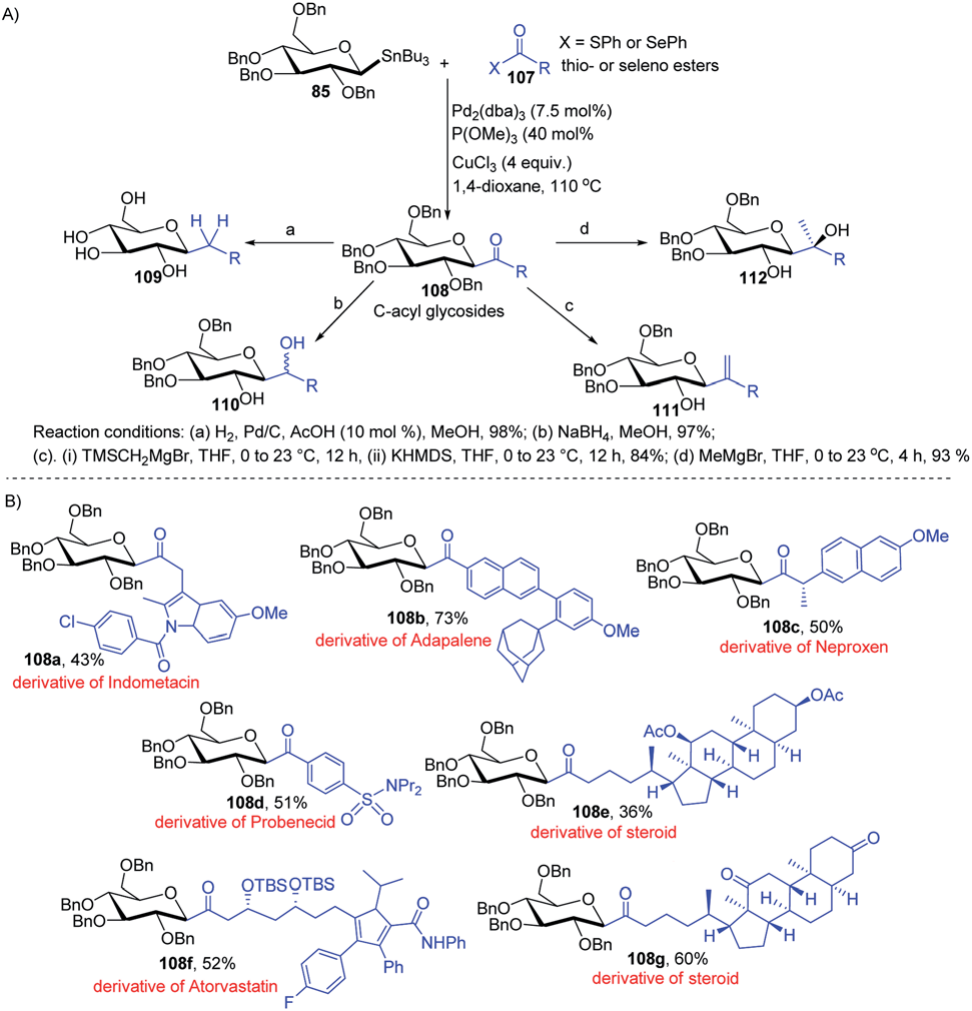
(A) Stille coupling with C(sp^2^)- and C(sp^3^)-thio (or seleno) esters and its applications (B) applications.

Having sufficient experience in dealing with anomeric glycosyl stannanes, Walczak's group proposed that replacing the hydrolytically less stable glycosidic bond with the hydrolytically stable C–C mimics is an excellent strategy to access glycomimetics that have improved physicochemical and pharmacological properties. In this endeavour, Walczak's group reported the synthesis of *C*-acyl glycosides (108) as synthetic scaffolds that can further be transformed into C(sp^3^)-linked and fluorinated glycomimetics 109–112*via* a stereo-retentive cross-coupling reaction of glycosyl stannanes (85) with C(sp^2^)- and C(sp^3^)-thio (or seleno) esters (107) as shown in (Scheme 30A).^70^ Key features of this protocol include (i) no deterioration of anomeric integrity and decarbonylation of the acyl donors and (ii) direct conversion of *C*1 ketones can introduce C(sp^3^)-glycomimetics into the anomeric position.

The versatility of Walczak's method is demonstrated by the simple conversion of a series of commercially available pharmaceuticals and other biologically active molecules like indomethacin, adapalone, probenecid, naproxen, atorvastatin and steroids into the thioesters, followed by cross-coupling with glycosyl stannane 85 into a series of bioactive acyl *C*-glycosides (108a–g) as depicted in (Scheme 30B).

### 2.14. *C*-Glycosides *via* intramolecular Stille-coupling

**Scheme 31:**
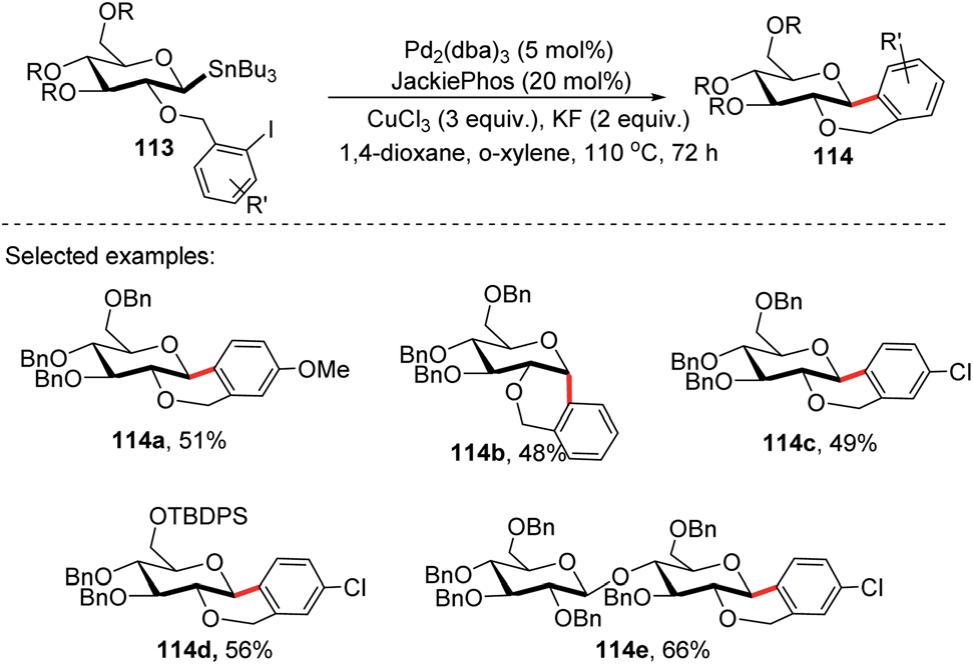
*C*-Glycosides *via* stereoretentive intramolecular Stille-coupling using aryl iodides.

On further expansion of their work towards the synthesis of *C*-glycosides of medicinal importance, Walczak's group developed an intramolecular version of glycosyl cross-coupling using aryl iodides for the synthesis of both *cis*- and *trans*-fused cyclic *C*-glycosides. The key factor is the stereoretentive transfer of the anomeric configuration from *C*-1 stannanes into a new C–C bond. The intramolecular glycosyl cross-coupling (Stille coupling) reaction between the nucleophilic anomeric carbon moiety and aryl halide moiety of substrate 113 produced *cis*- and *trans*-fused cyclic *C*-glycosides (114a–e) with high anomeric selectivity (Scheme 31).^71^

Securing high anomeric selectivity without compromising yield in an intramolecular version is more challenging than intermolecular cross-couplings. This is due to the increased probability of competing elimination reactions, the steric constraints developed on the C–Sn or C–Cu to C–Pd transmetalation and reductive elimination may hinder the C–C bond formation. In addition, steric crowding within the anomeric organo-palladium intermediate might also compromise the stereoretentive nature of the glycosyl cross-coupling reaction. Furthermore, kinetic analysis showed the secondary KIEs of 1.43 and 0.81 for intermolecular and intramolecular, respectively.^71^ This suggests different steric congestion at the transition state. It was further revealed that despite its high stereospecificity, intramolecular Stille coupling requires higher steric constraints at the anomeric carbon.

### 2.15. *C-*Glycosidic pseudo-disaccharides *via* the Sonogashira–Hagihara reaction

**Scheme 32:**
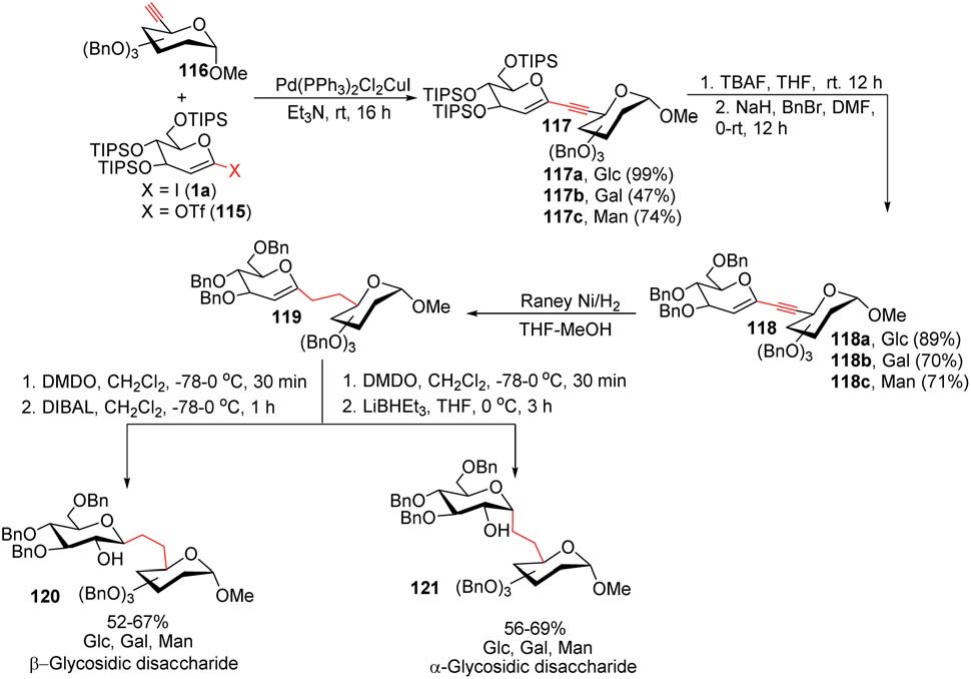
The Sonogashira–Hagihara reaction for the synthesis of α- and β-*C*-glycosidic pseudodisaccharides.

To address the problem of the *in vivo* instability of glycosidic bonds, the anomeric oxygen could be replaced by carbon so that carbohydrate mimetics called pseudodisaccharides (*C*-glycosidic disaccharides) are produced.^72^ In medicinal chemistry, the stability of such compounds against biological degradation makes their synthesis and biological evaluation an interesting journey.^73^ It was reported by Daniel B. Werz's group that a Pd-catalyzed Sonogashira–Hagihara coupling of a 1-iodoglycal (1a) or 1-triflatoglycal (115) with an alkynyl glycoside (116) generates glycosidic enyne systems (117a–c). Both α- and β-*C*-glycosidic disaccharides (120–121) can be produced by further conversion of the resulting enyne system (Scheme 32).^74^

### 2.16. *C*-Glycosides *via* Pd-catalyzed Ferrier-type rearrangement

**Scheme 33:**
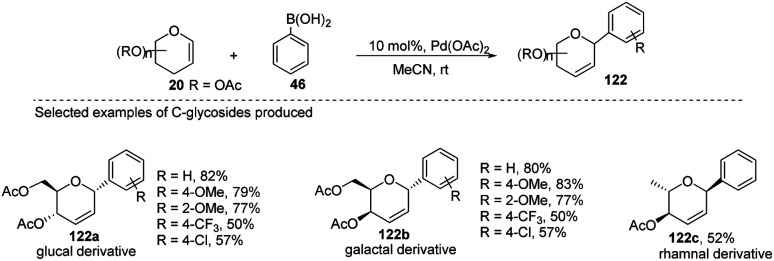
Pd-catalyzed carbon-Ferrier-type coupling for the synthesis of *C*-glycosides.

Maddaford *et al.* exploited Ferrier rearrangement for the synthesis of *C-*glycosides. The reaction involves the Pd(OAc)_2_-catalyzed addition of arylboronic acids (46) to glycals (20b). A carbon-Ferrier type product (122) was obtained *via* the *syn* addition of a σ–aryl-Pd complex to the glycal double bond, followed by the *anti-*elimination of Pd(OAc)_2_ as shown in (Scheme 33). This method provides a practical and convenient stereoselective synthesis of *C*-arylglycosides (122a–c, only selected examples shown). Due to the stability toward air and moisture and the wide range of boronic acid derivatives available, this method could have many useful applications in synthetic organic chemistry.^75^

**Scheme 34:**
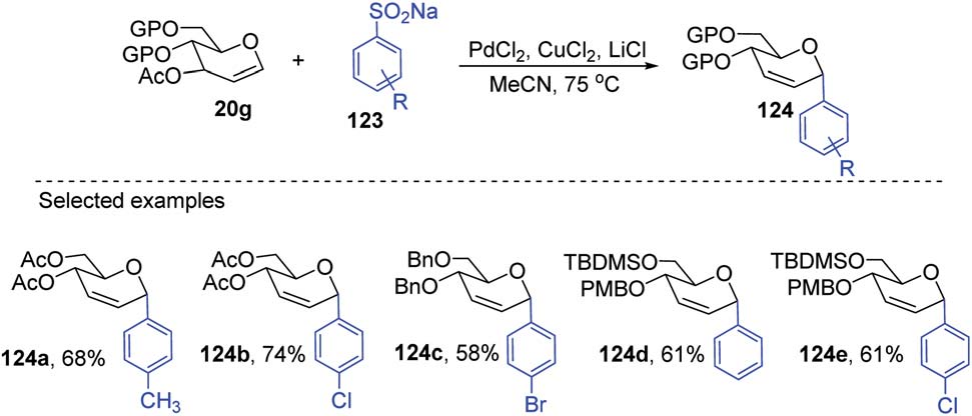
Synthesis of *C*-glycosides *via* Pd-catalyzed desulfitative Ferrier-type coupling.

Liu's group also employed a modified Ferrier rearrangement to synthesize 2-deoxy-*C-*arylglycosides. This strategy produced a series of *C*-aryl glycosides in moderate to good yields with excellent regioselectivity as well as stereoselectivity depending upon the configuration of the *C*-3 substituent of the glycals. This method is based on a Pd-catalyzed desulfitative Ferrier-type coupling of glycals (20g) and sodium arylsulfinates (123) to produce aryl-*C-*glycosides (124a–e) with 100% α-selectivity (Scheme 34).^76^

**Scheme 35:**
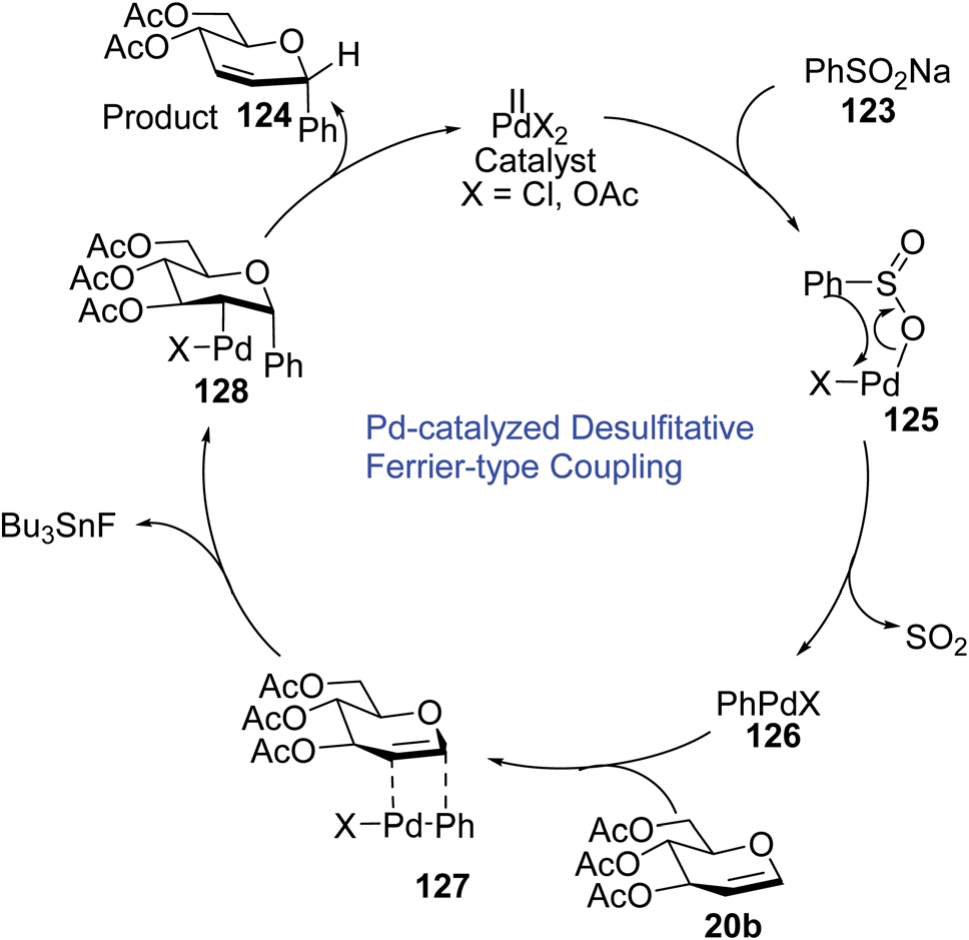
Mechanism of Pd-catalyzed desulfitative Ferrier-type coupling.

A plausible mechanism for the Pd-catalyzed desulfitative Ferrier-type coupling of glucal (54) with sodium benzenesulfinate (123) has been proposed based on observed stereoselectivities and literature reports (Scheme 35).^77–79^

The Pd(ii) catalyst exchanges ligands with benzenesulfinate (123) to generate the Pd complex (125), which in turn eliminates SO_2_ to produce aryl Pd(ii) species (126). The top side of the glucal is sterically hindered due to the *C*-3 substituent, therefore, the Pd(ii) species (126) attacks glucal (20b) from the bottom side to generate the π–Pd complex (127). *Syn* addition in species (127) leads to intermediate 128. In the final step, *C*-arylglycoside 124 is produced *via* the *anti* β-elimination of the acetyloxy group and the Pd(ii)-catalyst is regenerated.

### 2.17. *C-*Glycosides *via* the Pd-catalyzed S_N_2′-like reaction

**Scheme 36:**
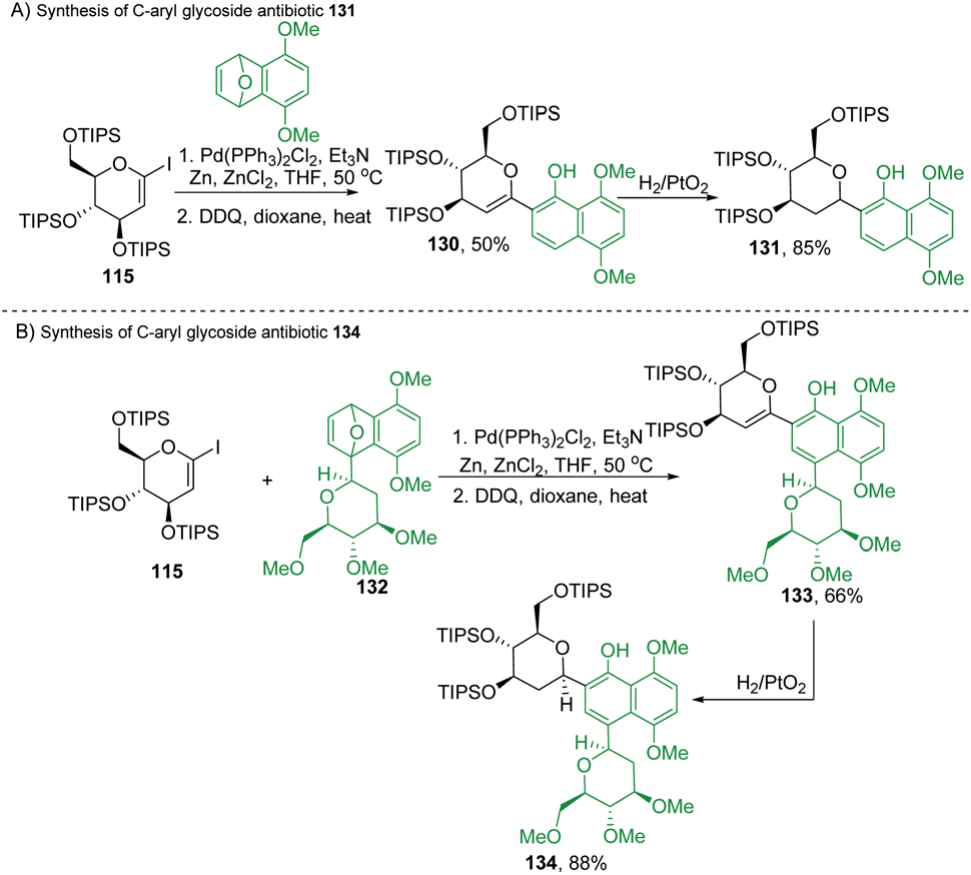
(A) Pd-catalyzed S_N_2′-like reaction ring-opening coupling (B) applications.

Martin's group synthesized biologically important *C-*glycoside antibiotics 131 and 134 through the Pd-catalyzed S_N_2′-like reaction (Scheme 36A and B). Martin *et al.* applied the procedure (optimized by Cheng *et al.*)^80^ to iodo-glycal (115) with cycloadduct (129) to produce a 1 : 1 mixture of the diastereomeric *cis-*dihydronaphthols 130, which on catalytic reduction produced the biologically significant antibiotic 131 (a *C-*glycoside) exclusively as a single stereoisomer as shown in Scheme 36A.^81^

Under similar conditions, the authors synthesized another *C*-aryl glycoside antibiotic 134. The Pd-catalyzed S_N_2′ ring-opening protocol was applied to iodoglycal 115 and cycloadduct 132 to produce dihydronaphthol, which gave 133 upon *in situ* oxidation in 68% overall yield. Catalytic hydrogenation of 133 reduced the glycal double bond to give the *C*-aryl glycoside antibiotic 134 (Scheme 36B).^81^

### 2.18. *C-*Glycosides *via* the dual catalysis approach

**Scheme 37:**
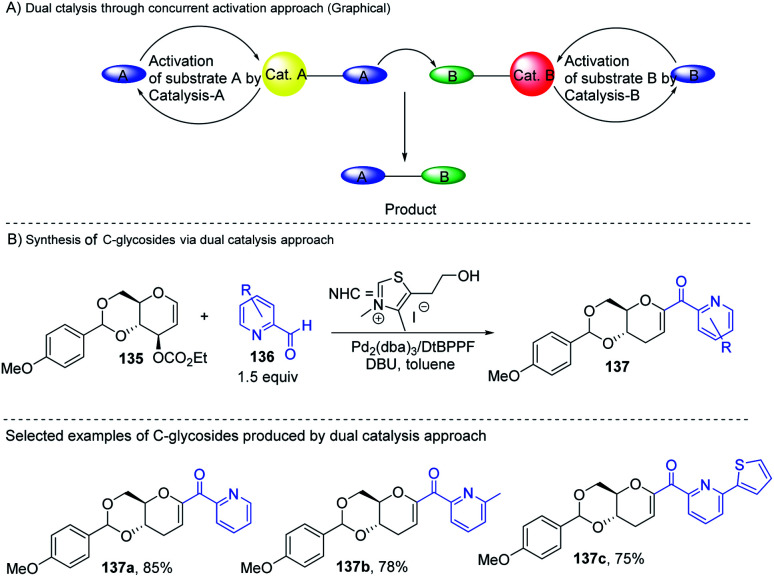
(A and B) Synthesis of *C-*glycosides; *via* the Pd- and NHC-catalyzed dual activation approach.

Liu's group reported novel co-operative catalysis through a dual activation approach (Scheme 37A) for the synthesis of *C*-glycosides using Pd complex and *N*-heterocyclic carbine (NHC) as dual catalysts. These two catalysts activated the two unreactive electrophilic reactants, glycals (135) and aldehydes (136), through the formation of a π–allyl Pd complex (138) and Breslow intermediate (139) to form *C*-glycosides (137) as depicted in Scheme 37B.^82^*N*-Heterocyclic carbine (5-(2-hydroxyethyl)-3,4-dimethylthiazolium iodide or NHC) catalyzed the aldehyde umpolung to form the Breslow intermediate to attack the electrophilic pi–allyl Pd complex, making this reaction successful.^83^ Liu's dual catalysis approach for the synthesis of *C*-glycosides has broadened the range of reacting partners for glycosylation reactions.

**Scheme 38:**
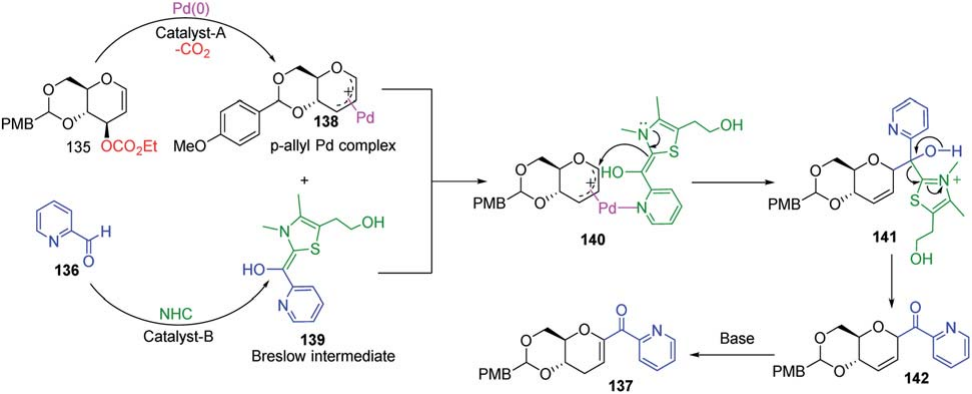
A plausible mechanism of the Pd- and NHC-catalyzed dual activation protocol.

A plausible mechanism has been proposed for this dual catalysis *C-*glycosylation methodology (Scheme 38). Pd(0)-catalyst activates glycal (135) to form the electrophilic π–allyl Pd-complex (138) while the second catalyst NHC activates aldehyde (136) to generate the Breslow intermediate (139). The intermediates 138 and 139 react through the N–Pd bond to form intermediate 140. The NHC-activated aldehyde part and allylic part of intermediate 140 undergo intramolecular nucleophilic addition to produce intermediate 141. This is followed by regeneration of the NHC catalyst and formation of *C*-glycoside 142, which rearranges to the final product under basic conditions 137 (Scheme 38).

### 2.19. *C-*Glycosides *via* C–H activation

**Scheme 39:**
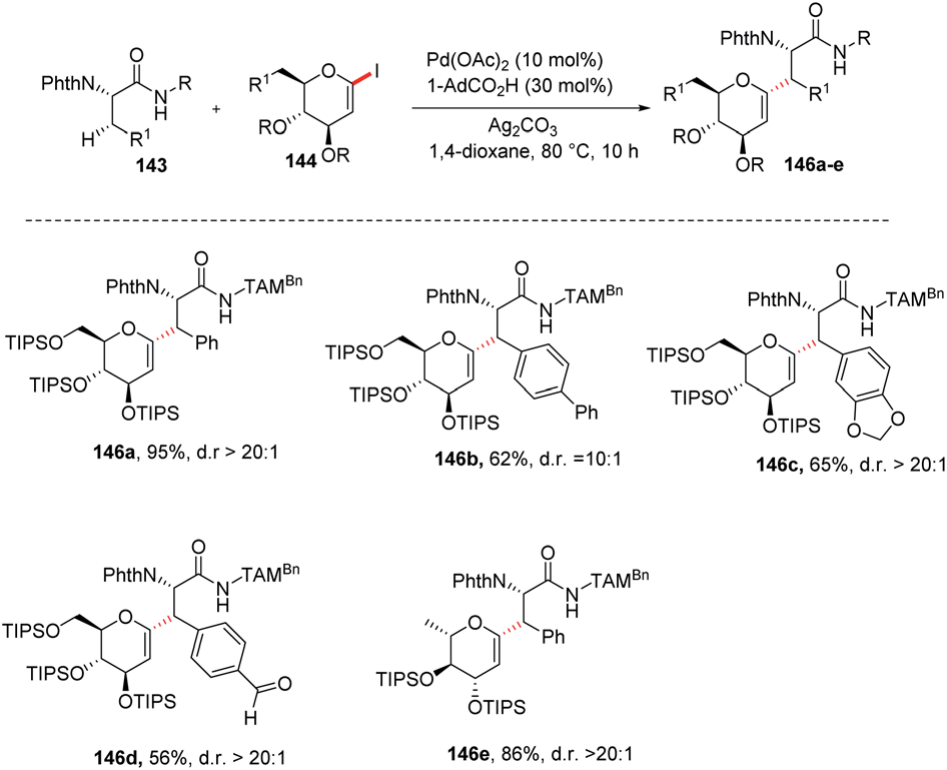
Synthesis of *C-*glycosides *via* palladium-catalyzed C(sp^3^)–H/C(sp^2^)–H glycosylation.

Jun Wu *et al.* reported a palladium-catalyzed C(sp^3^)–H/C(sp^2^)–H glycosylation method that is enabled by peptide isosteric click triazoles.^84^ This strategy is capable of forming the desired products with high levels of regio-, chemo- and diastereoselectivities. To explore the method, the preliminary optimization studies were carried out with challenging secondary C(sp^3^)–H glycosylation of triazolyldimethylmethyl (TAM) amide. It was noticed that in the presence of Pd(TFA)_2_ catalyst, and the AgOAc additive in dioxane, 60 °C was a less appropriate temperature but a slightly increased in temperature produced the desired product in 85% yield. However, on changing the AgOAc to Ag_2_CO_3_, the formation of glycopeptide in 95% yield was observed. On further screening and optimization, 1,4-dioxane was the best choice for solvent. To examine the versatility of this reaction, phenylalanine derivatives having different functional groups were checked and found to give the desired product in good yield with high diastereoselectivity (Scheme 39, 146a–e).

### 2.20. Directing group-assisted *C*-glycosylation

**Scheme 40:**
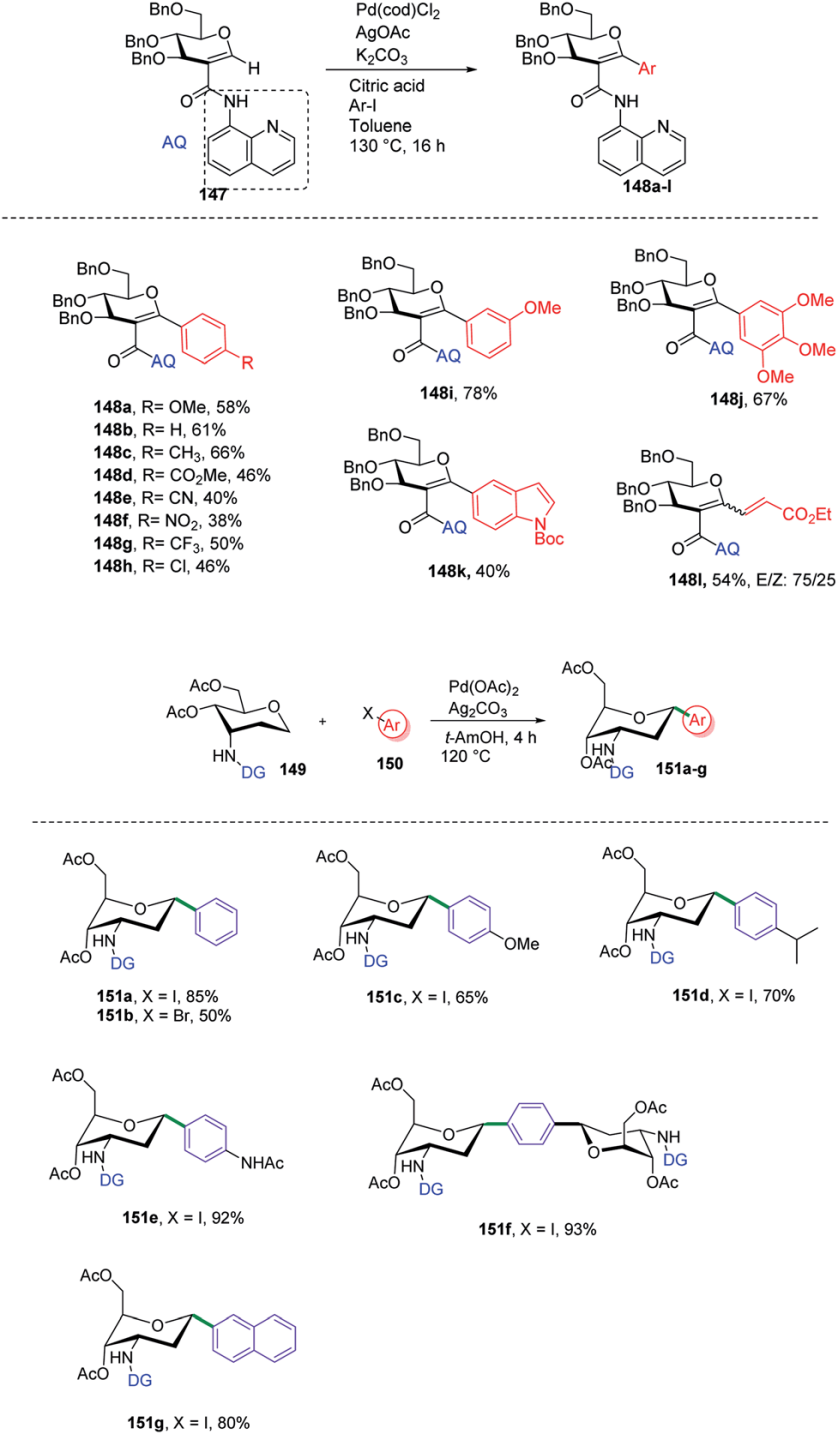
(A) Directing group-assisted C–H glycosylation. (B) Directing group assisted anomeric C–H activation.

*C*-Aryl glycosides are of great interest from the drug discovery point of view. There are several reported methods for the synthesis but these methods most often involve multiple steps, in addition to strong bases and bulkier ligands. Currently, C–H functionalization is of great interest but has its limitations of regioselectivity. Inspired by all these advantages and disadvantages, Angélique Ferry and co-workers reported^85^ a novel palladium-catalyzed strategy for the C–H functionalization of the anomeric position of *C*2-amidoglycals 147 for the synthesis of *C*-aryl/alkenyl glycosides. Initially, to set up the reaction conditions, previous amino carbonylation conditions were utilized with benzylated 2-iodo-d-glucal by taking 8-aminoquinoline as the amine partner, and the desired products were obtained in 85% yield. Further, C–H functionalization reactivity was tested with 4-iodoanisole (aryl partner) in the presence of a Pd catalyst and silver salt in toluene at 130 °C. Then, on screening the parameters it was noticed that an excess amount of weak base and a sub-stoichiometric amount of citric acid with Pd(cod)Cl_2_ are suitable reaction conditions that produced the desired product in 77% NMR yield and 58% in isolated yield. Further, this strategy was utilized in the synthesis of mono- and disaccharide glycosylated amino acids and dapagliflozin analogues (Scheme 40A, 148a–l).

*C*-(Hetero)aryl glycosides are an important class of carbohydrates that are utilized to replace the C–O bond with the strong glycosidic C–C bond. Naturally found *C*-aryl glycosides play a crucial role in many biological mechanisms. To date, several strategies *via* C–H bond activation have been reported but all these strategies selectively activate the β-C(sp^2^)–H bonds of groups, which are often in conjugation and are less effective for activating the less active C(sp^3^)–H bond. Inspired by this, Juba Ghouilem *et al.*^86^ reported a Pd-catalyzed strategy to synthesize *C*-(hetero)aryl glycosides with α-selectivity by activating the anomeric C(sp^3^)–H bond using a directing group at the *C*3 position. Initially, to execute the strategy, 3-amino sugar, having picolinic amide as a directing group at *C*3, was reacted with Pd(OAc)_2_, Ag_2_CO_3_ in *t-*AmOH at 120 °C and within 4 h, all the starting material was consumed and the desired product was obtained in 86% yield. Further, to optimize the conditions, a rapid examination of reagents and solvents was carried out, which revealed that Ag_2_CO_3_ is the best choice of base and toluene could be used in place of *t*-AmOH without affecting the yield of product. Inspired by the obtained good results, the examination of various substrates was performed. A wide range of sterically, electronically, *ortho*-, *meta*-, *para*-substituted aryl iodides and bromides were checked, and it was found that all the substrates are promising coupling partners to give *C*-aryl glycosides in moderate to good yield with controlled α-selectivity. Interestingly, it was observed that various reactive groups like –OAc, –NO_2_, –X (halogen), *etc.* survived, and also the sterically hindered group did not have any effect on the glycosylation process. Further, computational studies were carried out to determine the reason behind C-(sp^3^)-aryl bond formation, which revealed that the spatial arrangement of the directing group is responsible (Scheme 40B).

## 3. Pd-catalyzed 2*C*-branched sugar synthesis

### 3.1 From 2-haloglycals

**Scheme 41:**
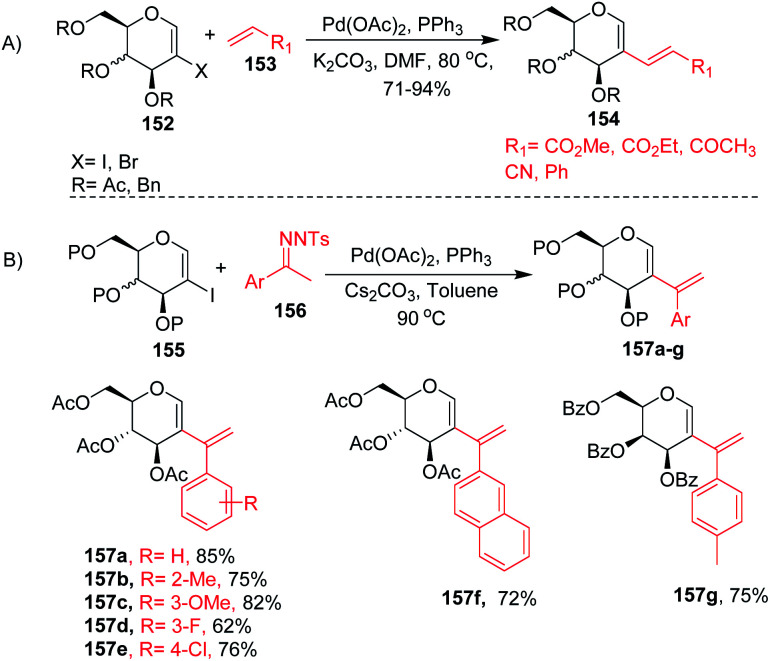
Cross-coupling reaction of 2-haloglycals with (A) terminal alkenes and (B) *N*-tosylhydrazones.

2-Haloglycals have been extensively used in the synthesis of 2*C*-branched sugars and annulated sugars. Vankar *et al.* developed the one-step synthesis of 2-haloglycals from glycals using NIS/AgNO_3_ and NBS/AgNO_3_ as reagent systems and the synthesized 2-haloglycals have been successfully utilized in the synthesis of 2*C*-branched sugars *via* the Heck coupling reaction (Scheme 41A).^87^ For the Heck coupling of 2-haloglycals with various terminal alkenes, various combinations of reagent systems were used and the most suitable combination was found to be Pd(OAc)_2_/PPh_3_, K_2_CO_3_ with DMF as a solvent. It was observed that various types of terminal alkenes, such as activated and unactivated, work well under the reagent system and provided the 2*C*-branched glycals in moderate to good yields. A similar type of cross-coupling strategy for 2*C*-branched sugars was developed by the Liu group^88^ by using *N*-tosylhydrazones as a source of coupling partners with various 2-iodoglycals (Scheme 41B). It was anticipated that various types of *N*-tosylhydrazones having both electron-withdrawing and electron-donating functionalities on the phenyl rings afford the desired 2*C*-branched sugars in moderate to good yields. The generality of the substrate scope was further extended with various glycals such d-galactal, d-xylal and glycals having different protecting groups.

**Scheme 42:**
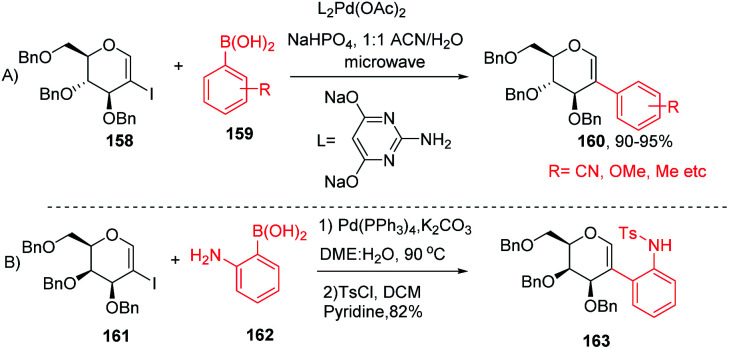
(A and B) Suzuki–Miyaura cross-coupling reactions of 2-iodoglycals.

The Davis group^89^ developed phosphine-free Suzuki–Miyaura cross-coupling reactions of 2-iodoglycals in aqueous medium for the synthesis of 2*C*-aryl glycosides. Various substituted phenylboronic acids, such as 4-methoxy, 2-methyl, 4-cyano, and 4-fluoro, reacted well with the 2-iodoglycals to afforded the respective desired products in excellent yields (Scheme 42A). The synthesized 2-*C*-aryl glycals were next transformed into 1,5-anhydro 2-*C*-aryl-2-deoxy alditols by the reduction of the endocyclic double bonds of glycals with Pd/C, H_2_ in methanol. Similarly, Rainier and co-workers^90^ further utilized the 2-iodo-tri-*O*-benzyl-d-galactal for the synthesis of indoline and benzofuran by using Suzuki–Miyaura cross-coupling reactions and oxidative cyclization. It was demonstrated that when 2-iodo-galactal 161 reacted with 2-amino phenylboronic acid 162 under Pd catalysis by using DME:H_2_O as a solvent the desired 2*C*-aryl galactal 163 was obtained, which was further subjected to oxidative cyclization with NBS/*m*-CPBA to obtain the respective *cis*/*trans* indolines in good yields.

**Scheme 43:**
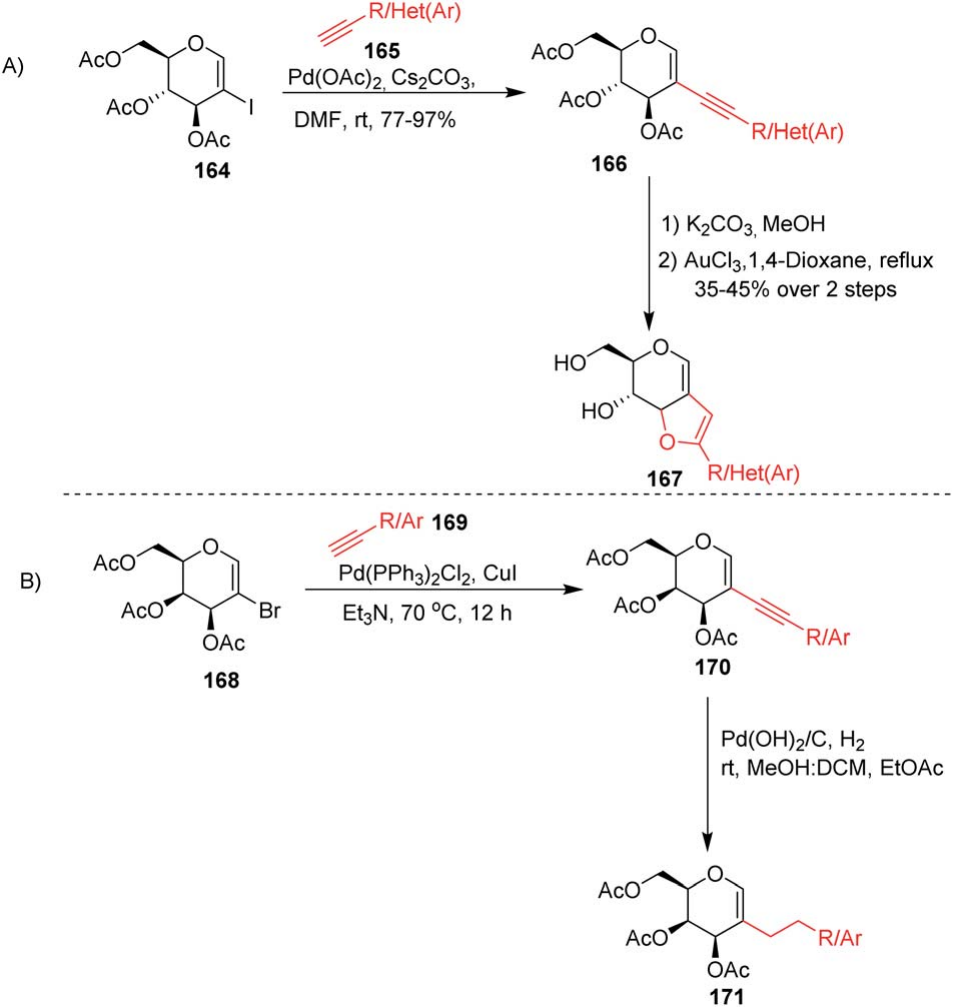
Sonogashira–Hagihra cross-coupling reactions of 2-haloglycals.

Stefani and his co-workers^91^ utilized the Sonogashira–Hagihra cross-coupling reaction for the synthesis of 2-alkynyl-glucal derivatives 166 under ligand- and copper-free conditions (Scheme 43A). It was found that a variety of aliphatic, aromatic, and heterocyclic acetylenes, when reacted with 2-iodo-3,4,6-tri-*O*-acetyl-d-glucal 164 using Pd(OAc)_2_ and Cs_2_CO_3_ in DMF at room temperature, gave the respective *C*-2 alkynyl derivatives of d-glucal in good to excellent yields. Later on, the sugar-fused furan derivative 167 was synthesized by using 2-alkynyl-d-glucal derivatives 166 under AuCl_3_-catalyzed cyclization. Werz and his group^92^ similarly utilized the Sonogashira–Hagihra cross-coupling reactions by using 2-bromogalactals 168 and observed that the 2-bromogalactals were less reactive as compared to the 1-iodoglycals. The authors assumed that the electron density at the *C*-2 position of glycals is high and that the 2-palladated glycal is a weak electrophile for electronically poor or neutral alkynes. To get the desired product, elevated temperature was required and the desired products 170 were obtained in moderate yields. Pearlman's catalyst (Pd(OH)_2_/C) was used for the reduction of triple bonds of alkynes to get 2*C*-alkylated sugars 171 (Scheme 43B).

**Scheme 44:**
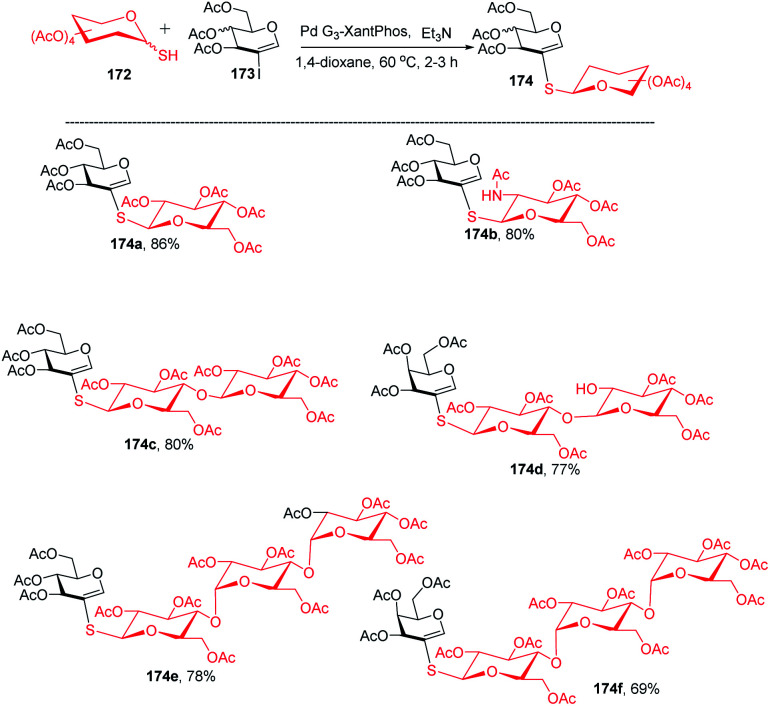
Buchwald–Hartwig–Migita cross-coupling of 1-thiosugars with 2-iodoglycals.

The Buchwald–Hartwig–Migita cross-coupling of 1-thiosugars 172 with 2-iodoglycals 173 was achieved by the Messaoudi group^93^ by using the Pd-G3 XantPhos palladacycle precatalyst. In this method, various 1-thiosugars 172 such as α- or β-mono-, di-, and polythiosugar derivatives have been utilized to efficiently synthesize a series of (1→2)-*S*-linked thiosaccharides and *S*-linked glycoconjugates 174 (Scheme 44). It was observed that the nature of the glycal partners does not interfere with the outcome of the reaction and works well with various 2-iodoglycals such as 2-iodoglucal and galactal derivatives. It was also observed that all the reactions proceeded with the retention of the anomeric configuration with good yields (Scheme 44).

**Scheme 45:**
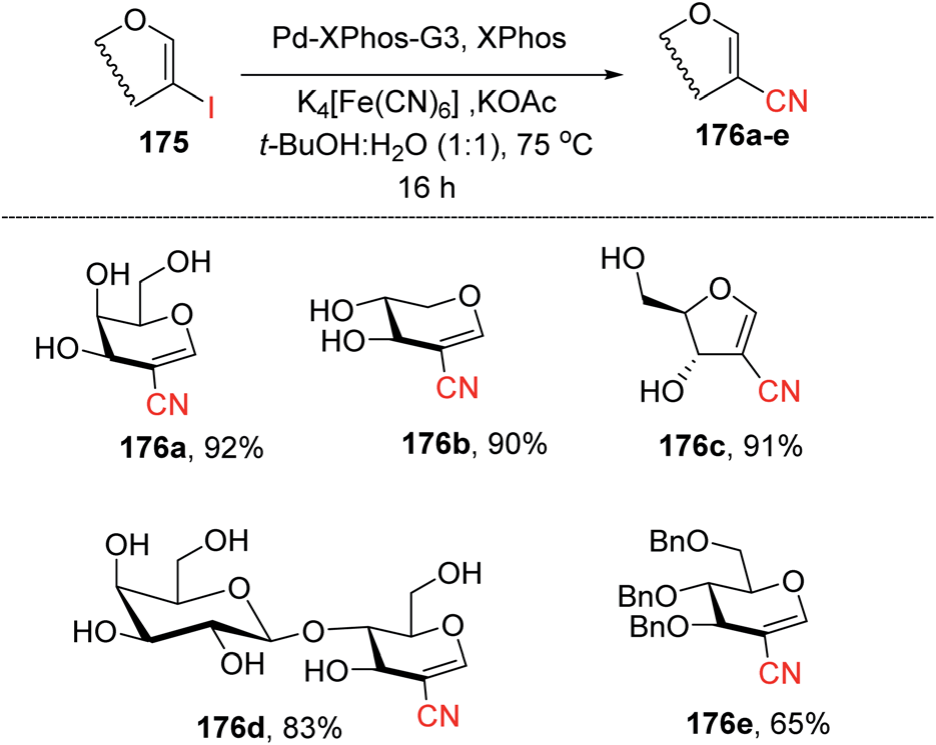
Pd-catalyzed cyanation of 2-iodoglycals.

Ferry and co-workers^94^ developed a mild palladium-catalyzed cyanation of unprotected 2-iodoglycals in aqueous media to generate a library of diverse 2*C*-branched glycoconjugates (Scheme 45). This method worked in aqueous medium and with unprotected 2-iodoglycals by using K_4_[Fe(CN)_6_] as a source of CN. It was found that the use of co-solvent *t*-BuOH along with 1,4-dioxane is crucial for the reactivity of the reaction and the best results were obtained when the solvent ratio was 1 : 1. The substrate scope of the reaction was further extended with various protected and unprotected glycals such as d-xylal, d-galactal, l-rhamnal, furanoside glycal and some disaccharide type 2-iodoglycals (Scheme 45, 176a–e).

**Scheme 46:**
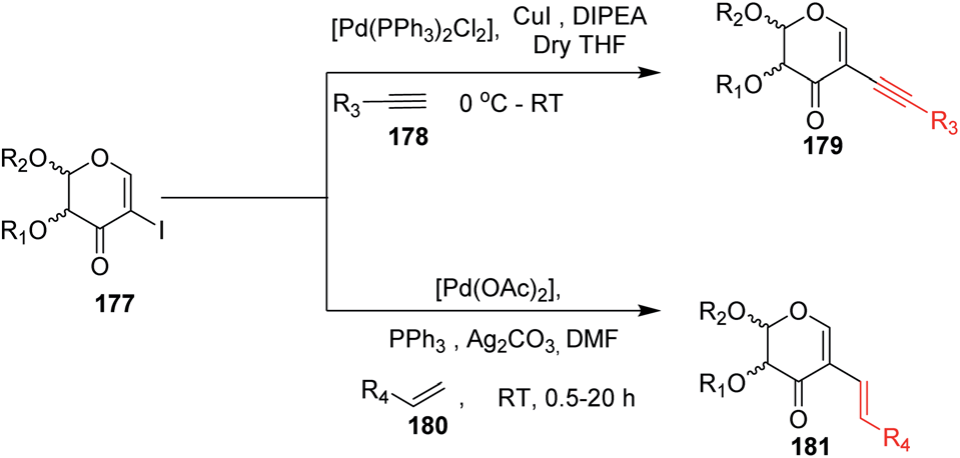
Transformations of 2-iodo-enones under Pd-catalysis.

Das and co-authors^95^ utilized 2-iodo-enones derived from glycals under palladium-catalyzed Sonogashira and Heck coupling with acetylenes and alkenes for the generation of 2*C*-alkynyl and akenyl glycals-enones (Scheme 46). The synthesized 2*C*-branched enones were later transformed into highly chirally enriched furans by AuCl_3_ and NBS-catalyzed cyclization followed by aromatization reactions.

**Scheme 47:**
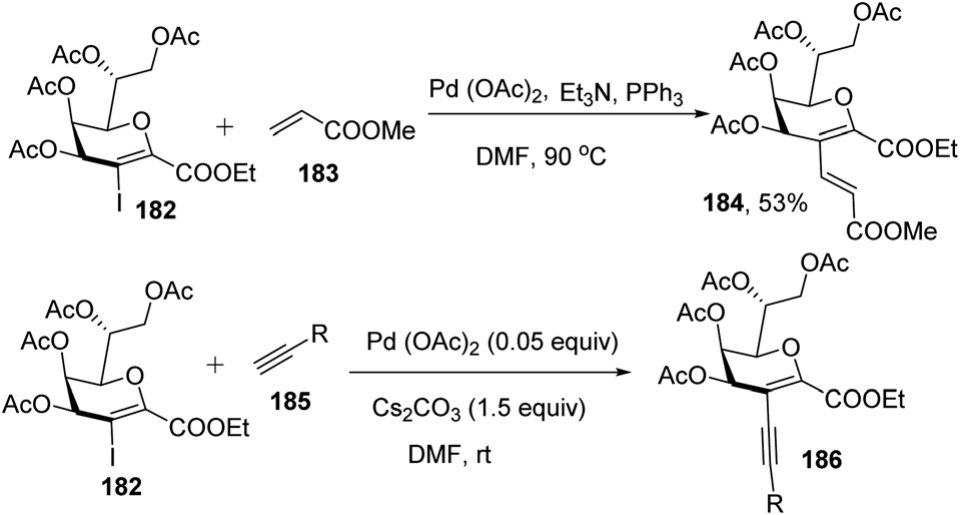
Transformations of 3-iodo-Kdo-glycals under Pd-catalysis.

Kdo sugars are an important component of lipopolysaccharides (LPS) found in the outer membrane of Gram-negative bacteria. The lipid A of the LPS is connected to two Kdo units forming lipid A-Kdo_2_, which is essential for the integrity of the outer membrane and for cell viability. Inhibiting Kdo transferases (WaaA) involved in lipid A-Kdo_2_ biosynthesis is, therefore, an exquisite target for antibiotics. Yang and co-workers^96^ developed a cross-coupling methodology for the functionalization of the 3-iodo-Kdo-glycals 182 with terminal alkenes 183 and acetylenes 185 under Pd catalysis for the synthesis of 3-*C*-branched 3-deoxy-d-mannooct-2-ulosonic acid (Kdo) analogues (Scheme 47, 184, 186). The substrate scope of the reaction was further enhanced with variety of terminal akenes and acetylenes.

**Scheme 48:**
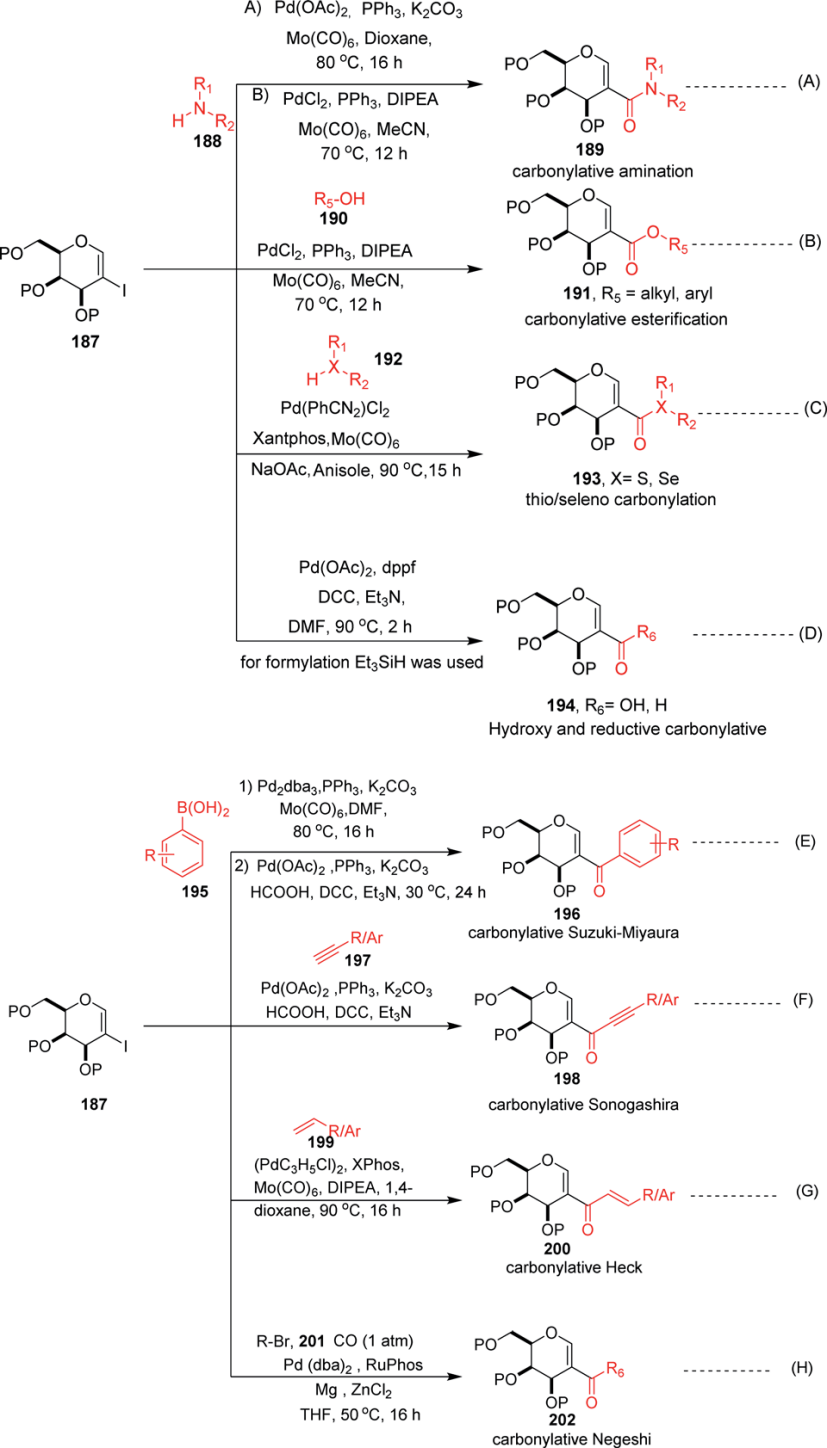
(A) Carbonylation reactions of 2-haloglycals with various coupling partners. (B) Carbonylation reactions of 2-haloglycals with various coupling partners.

Carbonylation reactions under transition metal catalysis have become a widely used area of research in organic synthesis throughout recent decades. The insertion of CO into an organic moiety introduces a carbonyl group as well as the addition of one carbon to an organic compound. The carbonyl group is one of the most common functionalities in bioactive compounds. Pd-catalyzed carbonylation reactions have been well explored by using 2-haloglycals with different coupling partners for the generation of 2*C* branched glycoconjugates (Scheme 48). Nadège Lubin-Germain *et al.*^97^ and Hélio A. Stefani^98^ developed amino carbonylation reactions with 2-iodoglycals under Pd catalysis. A stoichiometric amount of metal carbonyl Mo(CO)_6_ was used as a solid carbonyl source in the reaction medium. It was noticed that a variety of amines reacted well under the developed reagent system and affords the library of 2*C*-branched glycoconjugates (Scheme 48A). Hélio A. Stefani and his group later on extended their strategy for the synthesis of 2*C*-branched glucal esters while replacing amines with alcohols under the same optimized reaction conditions (Scheme 48B). Thio and seleno carbonylation reactions of 2-iodoglycals were developed by Hélio A. Stefani and co-workers^99^ for the synthesis of 2-*C*-branched thio and seleno esters of glycals (Scheme 48C). Pd(PhCN)_2_Cl_2_ in combination with xantphos as a ligand under basic medium provided the respective thioester of glucal in excellent yield. The substrate scope was shown with a variety of aromatic and aliphatic thiols and moderate to good yields were obtained for all the thioesters of glucals. Later on, by replacing thiols with selenols under the standardized reaction conditions, the seleno ester of glycals were formed in moderate to good yields. Mukherjee *et al.*^100^ synthesized 2-formyl and 2-carboxylic acids of glycals by using formic acid as a carbonyl source under Pd catalysis. Formic acid in combination with DCC released one molecule of CO for the carbonylation reaction in the reaction medium. In this strategy, various 2-iodoglycals with different protecting groups survived and provided the desired carbonylative products in moderate to good yields. Triethylsilane was used as a source hydride in the reductive carbonylation reaction for the synthesis of 2-formyl glycals (Scheme 48D).

Carbonylative Suzuki–Miyaura cross-coupling reactions of 2-haloglycals with different boronic acids were explored independently by Angélique Ferry and co-workers,^101^ along with Mukherjee *et al.*,^102^ by using different carbonyl precursors such as Mo(CO)_6_ and HCOOH. In both the strategies, it was found that the developed reagent system worked well with various 2-haloglycals 187 and boronic acids 195 (Scheme 48E). Mukherjee *et al.* later on extended their strategy to the synthesis of 2*C* branched alkynones *via* carbonylative Sonogashira coupling (Scheme 48F). α,β-Unsaturated 2-ketoglycosides *via* a Pd-catalyzed carbonylative Heck reaction of 2-iodoglycals was developed by Hélio A. Stefani and co-workers^103^ by using 2-iodoglycals with various terminal alkenes (Scheme 48G). In this approach Mo(CO)_6_ was used in stoichiometric amounts as a solid CO source, along with Pd as the catalyst and XPhos as the ligand. The substrate scope of the reaction was explored and moderate to good yields were observed with olefins bearing electron-neutral and electron-rich groups (Scheme 48G). In continuation of their efforts on carbonylation reactions, Helio A. Stefani and co-authors^104^ checked the reactivity of unactivated alkyl halides *via* Negishi type cross-coupling reactions with 2-iodo glycals under Pd catalysis by using carbon monoxide gas as a source of CO (Scheme 48H). A library of carbonyl-inserted compounds was generated with a variety of alkyl halides. It was found that by exchanging the alkyl halides with aryl halides as a coupling partner with 2-iodoglycals, the carbonylative products were also obtained in moderate to good yields. Next, the reactivities of vinylic alkyl halides were checked and found to be unreactive under the reaction conditions. It was also observed that different sugar partners having different groups were compatible under the reaction conditions.

### 3.2 From glycals *via* C–H activation

**Scheme 49:**
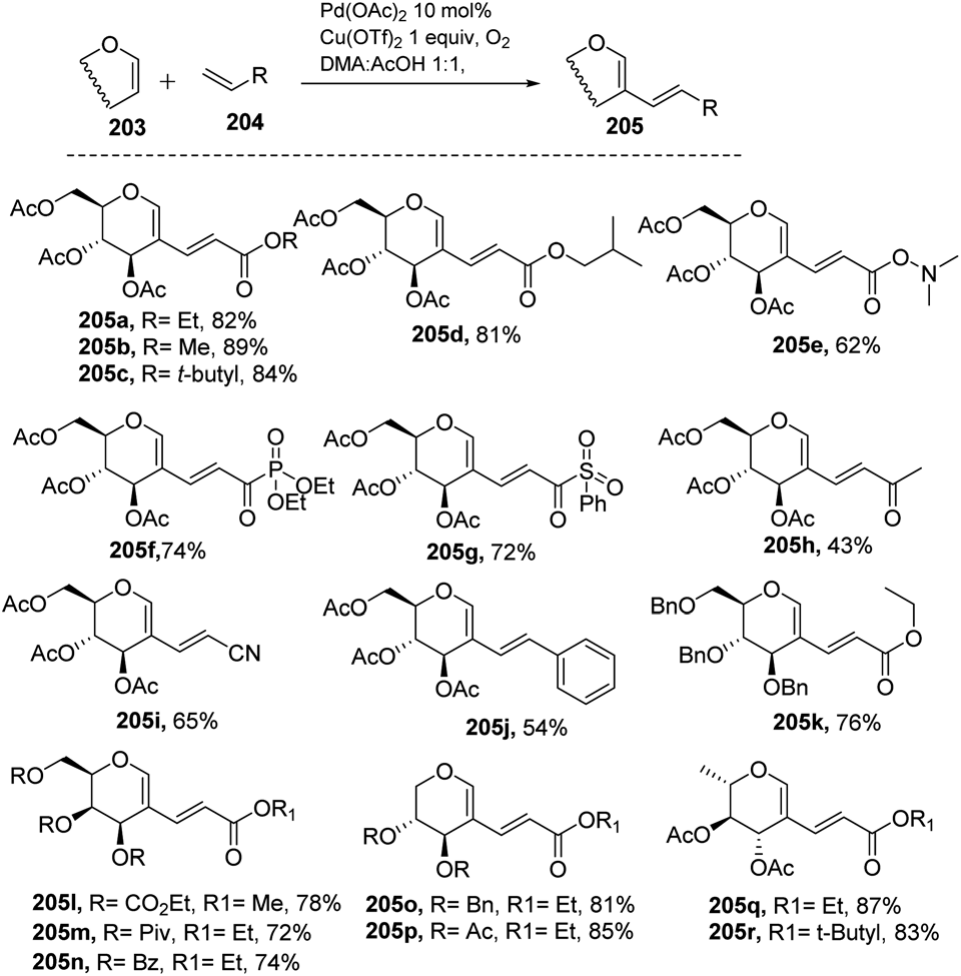
Direct coupling of protected glycals with activated alkenes.

Liu *et al.* in 2011^105^ developed a method for the alkenylation of glycals using the direct *C*-2 position by activating the Csp^2^–H bond under palladium catalysis (Scheme 49). The reaction required Pd(OAc)_2_, Cu(OTf)_2_, 1 atm of O_2_ to complete the catalytic cycle. The substrate scope of the reaction was enhanced with different types of protected glycals and activated alkenes (Scheme 49, 205a–r). However, the authors failed with unprotected glycals, glycals having acid-labile protecting groups and unactivated alkenes.

**Scheme 50:**
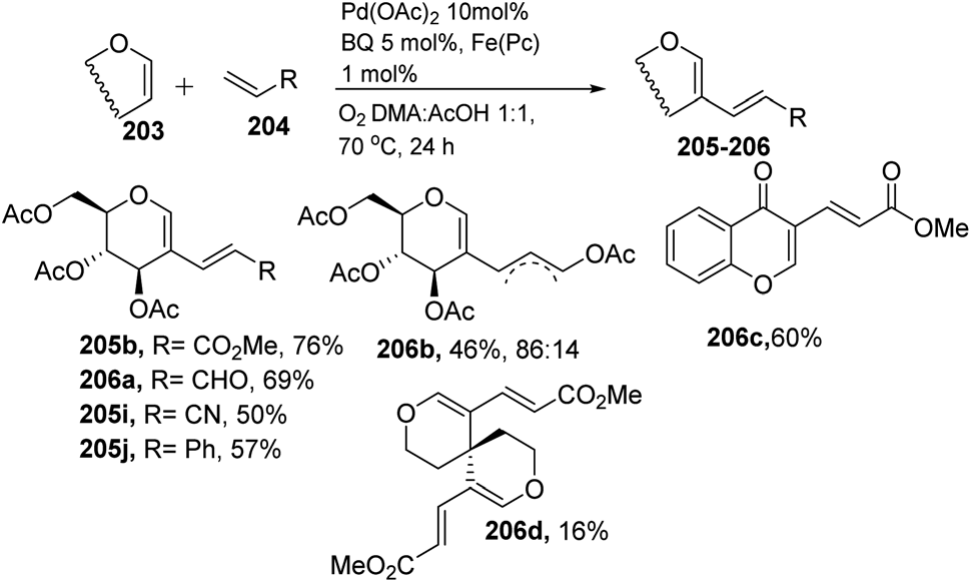
Biomimetic aerobic oxidative coupling of enol-ethers with various alkenes.

In 2013, Backvall^106^ developed a detailed synthetic strategy for the synthesis of conjugated dienes *via* a biomimetic aerobic oxidative coupling of two C-vinyl–H bonds (Scheme 50). In previous literature reports, the alkenylation of glycals *via* C–H bond activation was achieved with a heavy loading of palladium catalyst and stoichiometric amounts of oxidant and bases (Cu(ii), Ag(i), peroxides, *etc.*). Backvall *et al.* reported a more general strategy with low catalyst/oxidant loading for the catalytic cycle and the use of molecular oxygen plays a pivotal role in this transformation. Catalytic quantities of p-BQ and Fe(Pc) as electron-transfer mediators and O_2_ at atmospheric pressure can regenerate the catalyst in its active form.

**Scheme 51:**
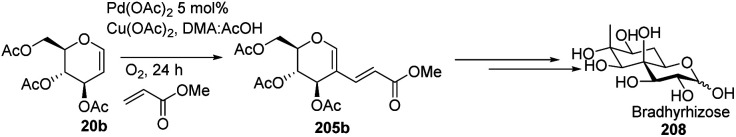
Synthesis of bradyrhizose using *C*-2 alkenyl d-glucal.

In 2015, Biao Yu and his group^107^ modified the previously reported work by Liu *et al.* and prepared *C*-2 alkenyl d-glucal on the gram scale. Further, they utilized this substrate for the synthesis of bradyrhizose, a unique inositol-fused monosaccharide relevant to a Nod factor independent nitrogen fixation (Scheme 51).

**Scheme 52:**
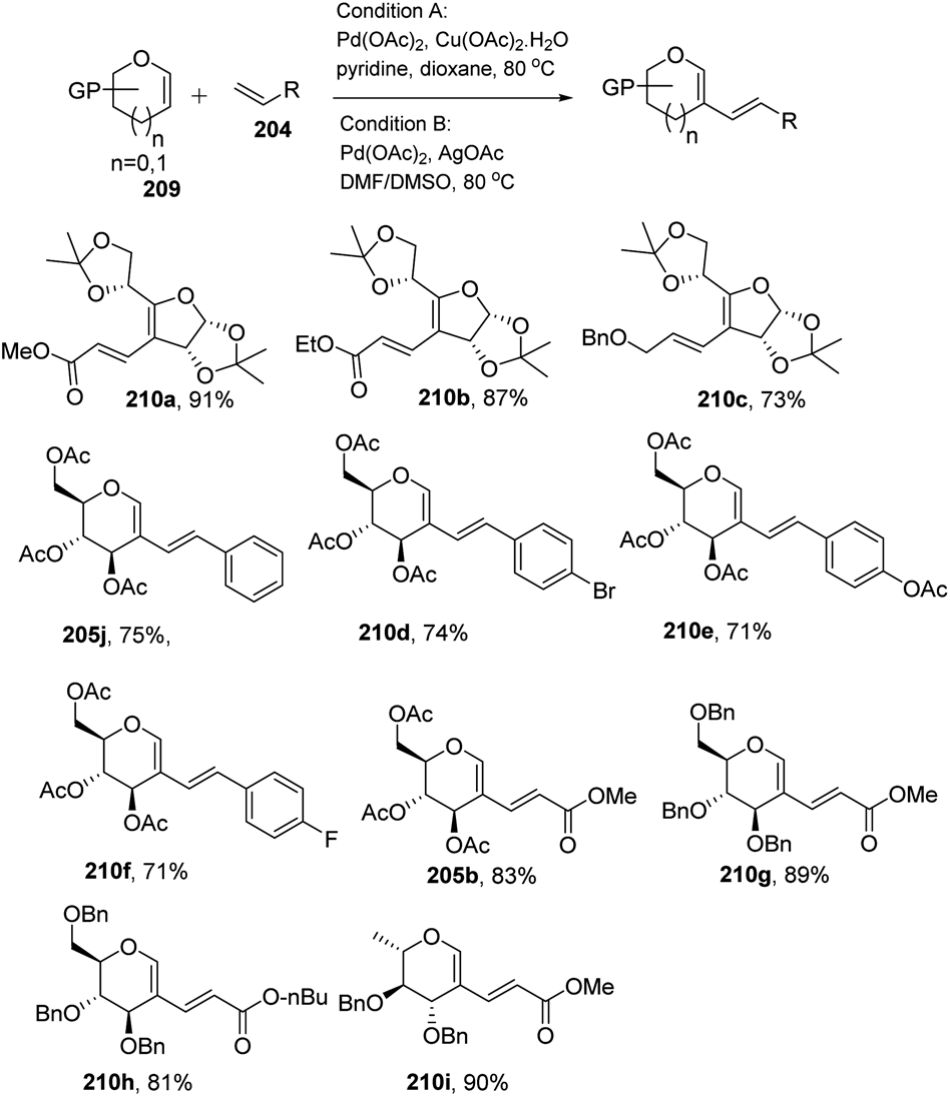
Synthesis of *C*-2/*C*-3 alkenyl sugars *via* C–H activation.

**Scheme 53:**
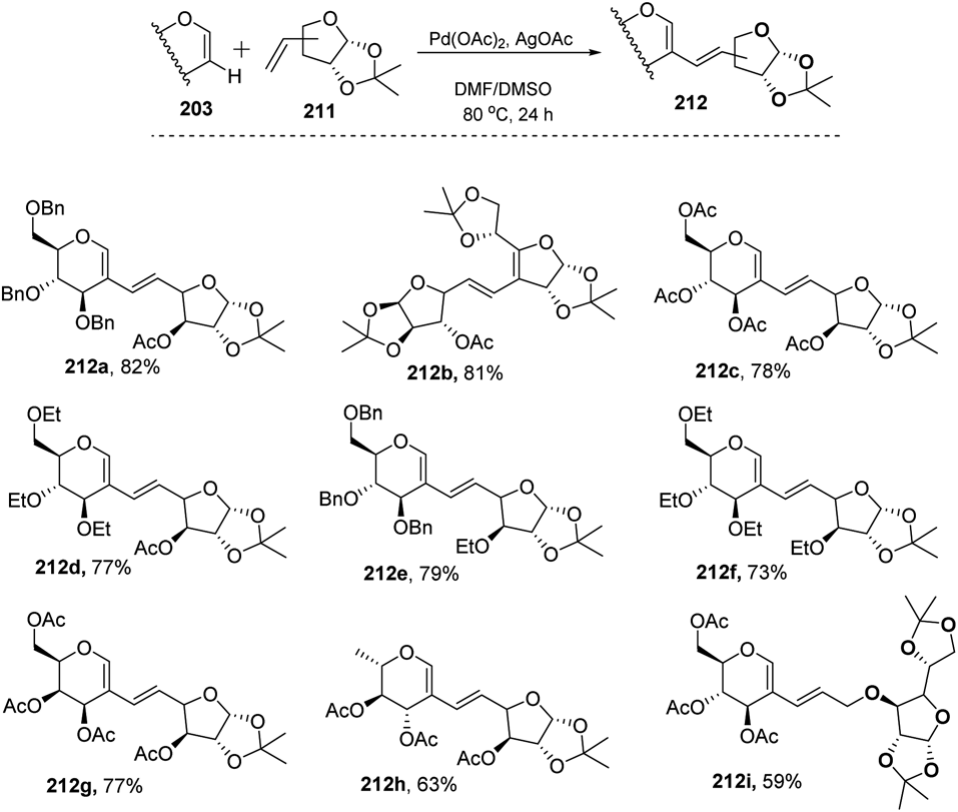
Synthesis of ethylene-linked pseudodisaccharides.

In most of the literature discussed so far, the developed reaction conditions work for only activated alkenes and in some cases with unactivated alkenes along with a mixture of different products or very poor yield. In 2018 Mukherjee *et al.*^108^ developed a very mild reagent system in which unactivated alkenes like styrenes, terminal sugar-derived alkenes and most importantly, the different protecting groups of sugars survived under the reagent system and provided the desired products in moderate to good yields (Scheme 52). The strategy was successfully applied for the synthesis of different substituted sugars like *C*-2, *C*-3 and *C*-6 branched with various activated and unactivated olefins. Most of the studies focused on the utilization of aromatic and aliphatic alkene sources but there is no such procedure in which sugar-based terminal alkene sources have been used for the construction of pseudodisaccharides, which otherwise requires multi-steps with very poor overall yields. A series of sugar-based terminal alkenes have been used with various differently protected glycal derivatives for the generation of ethylene-linked pseudodisaccharides (Scheme 53).

**Scheme 54:**
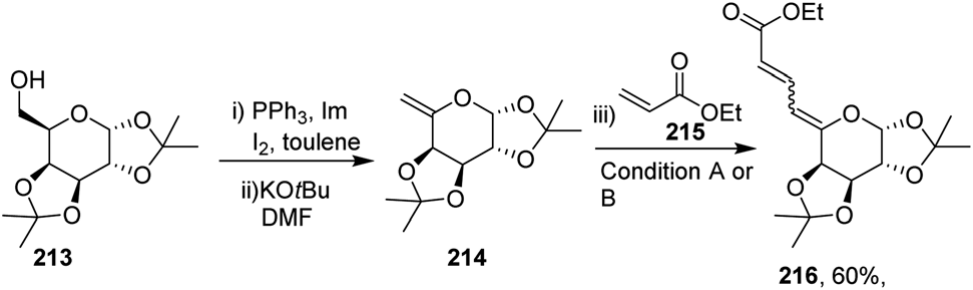
Coupling of exoglycals with ethyl acrylate.

After getting successful results in the preparation of various dienes with endoglycals, the same group utilized the exoglycal 214 as a coupling partner with terminal alkene 215. Exoglycal 214 was prepared from diacetone-d-galactose 213 coupled with ethyl acrylate 215 under optimized reaction conditions to obtain the desired diene 216 in good yield (60%) as a mixture of *trans*, *anti*, *cis* and *trans*, *anti*, *trans* (*E*,*anti*,*Z*:*E*,*anti*,*E*) as determined from matching the *J* values (Scheme 54).

**Scheme 55:**
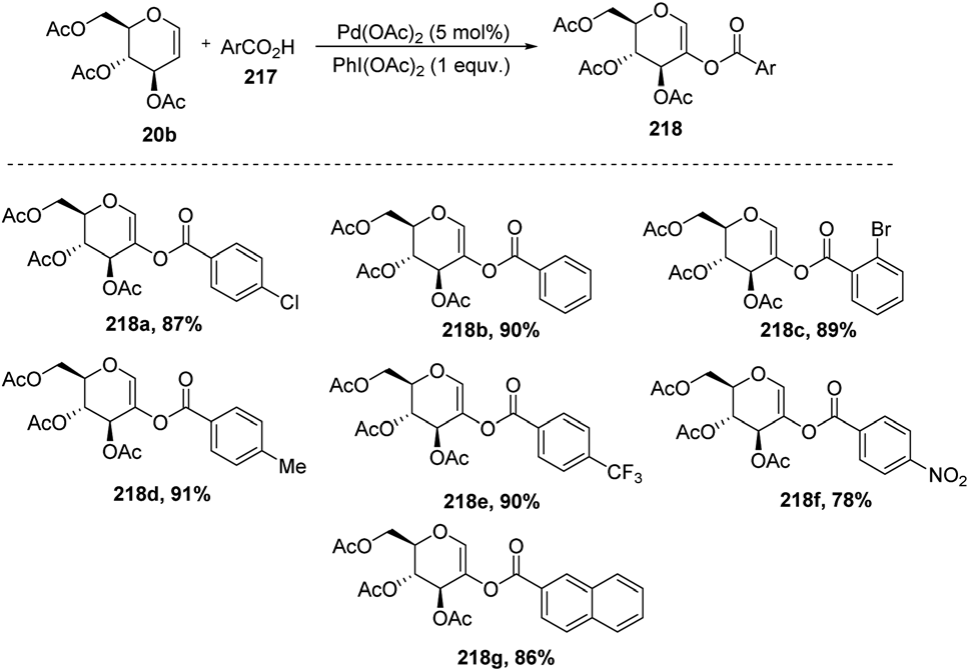
Coupling of glycals with aromatic acids under Pd catalysis.

In 2009, Zubeda Begum *et al.*^109^ demonstrated the *C*-2 functionalization of glycals through oxidative cross-coupling with an aromatic carboxylic acid. In this approach, when tri-*O*-acetyl-d-glucal 207 reacted with aromatic acids 217 in the presence of Pd(OAc)_2_ and PhI(OAc)_2_, *C*2-acyloxyglycals 218 were formed and the synthesized compounds were further utilized for biologically active natural product synthesis (Scheme 55).

**Scheme 56:**
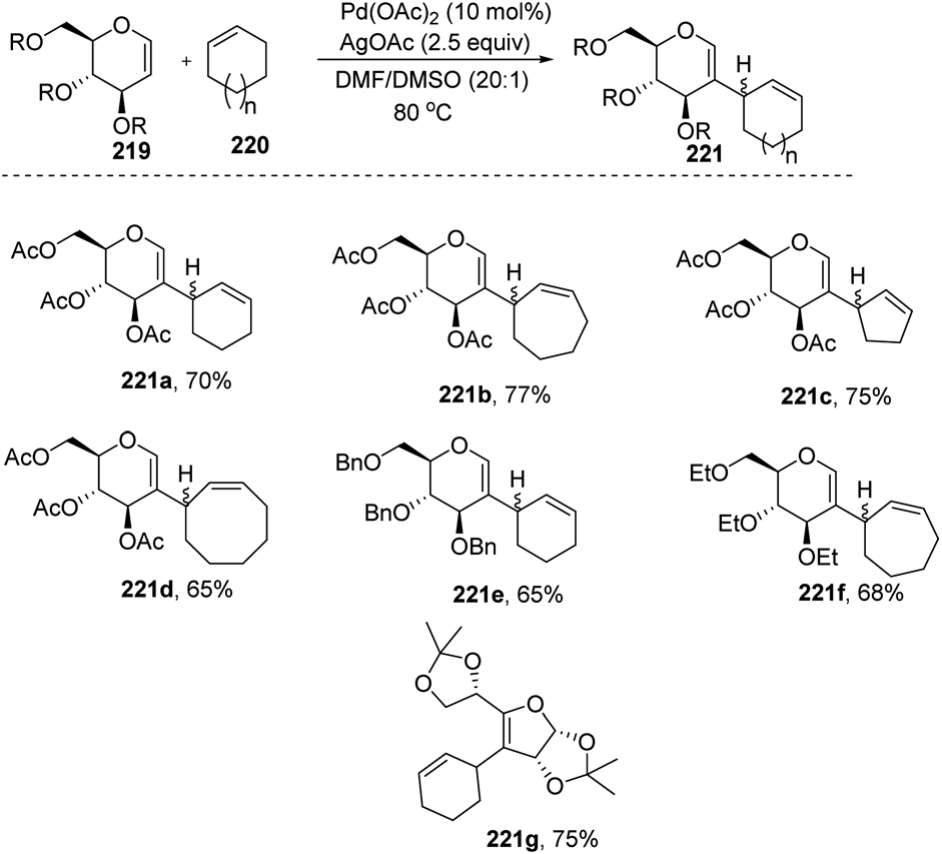
Substrate scope for the allylation of glycals with cycloalkenes.

Mukherjee *et al.*^110^ demonstrated that sugar enol ethers undergo efficient coupling at the *C*-2 position with unactivated cycloalkenes under a low Pd loading and afforded the allylic substituted products (Scheme 56). High diastereoselectivity was observed at the allylic center with sterically hindered substrates (221g). The generation of a π–allyl complex by the Pd(ii) catalyst *via* cleavage of the allylic C–H bond of the cycloalkene may be responsible for the formation of sp^2^–sp^3^ coupling products. The substrate scope of the reaction was checked with various cyclic alkenes and glycals with different protecting groups.

**Scheme 57:**
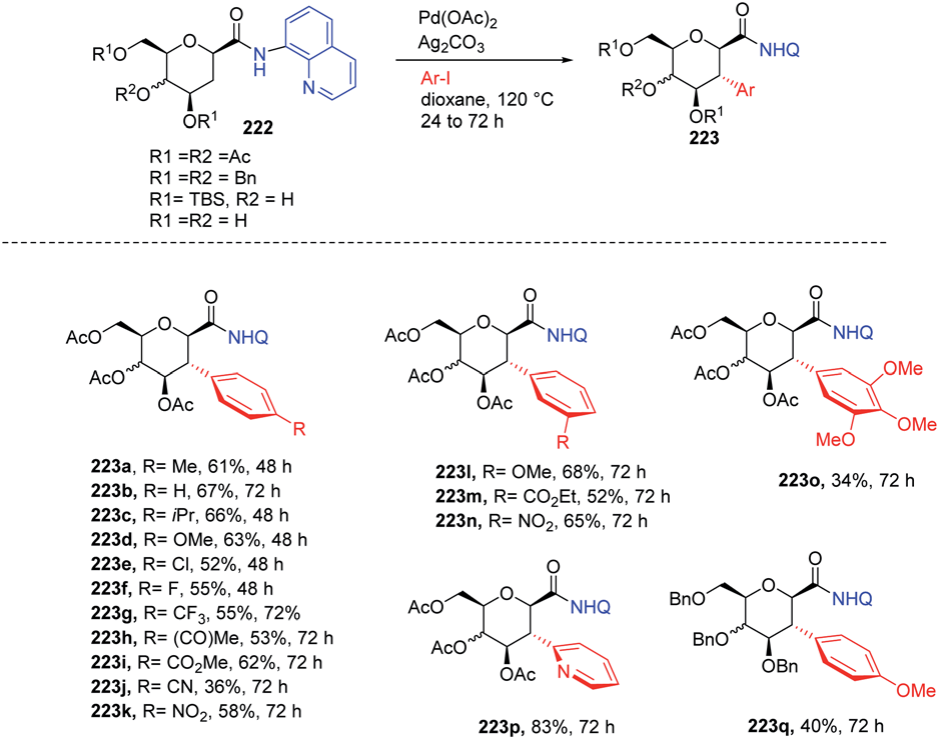
Diastereoselective *C*-2 arylation glycosides.

Nicolas Probst *et al.*^111^ reported the Pd-catalyzed diastereoselective C(sp^3^–H) arylation of glycosides. A series of β- and α-glycosides was checked and proved to undergo selective Pd-catalyzed coupling with different aryl iodides to make a library of functionalized 3-arylglycosylamides. To execute the diastereoselective C(sp^3^–H) arylation method, the optimization of the reaction parameters was carried out by utilizing aminoquinoline-directing β-glycoside as the standard substrate. The screening of parameters indicated that Pd(OAc)_2_ (20 mol%), Ag_2_CO_3_ (2 equiv.), dioxane (0.1 M), 120 °C and N_2_ conditions are best suited to the reaction and the desired product was obtained in high yield. It was also observed that the reaction did not work with β-glycoside having perfluoro toluidine and amino methylpyridine as the directing group. Interestingly, this strategy selectively exhibited 2,3-*trans* arylation for the first time and mechanistic studies were further elucidated by the DFT calculations (Scheme 57).

**Scheme 58:**
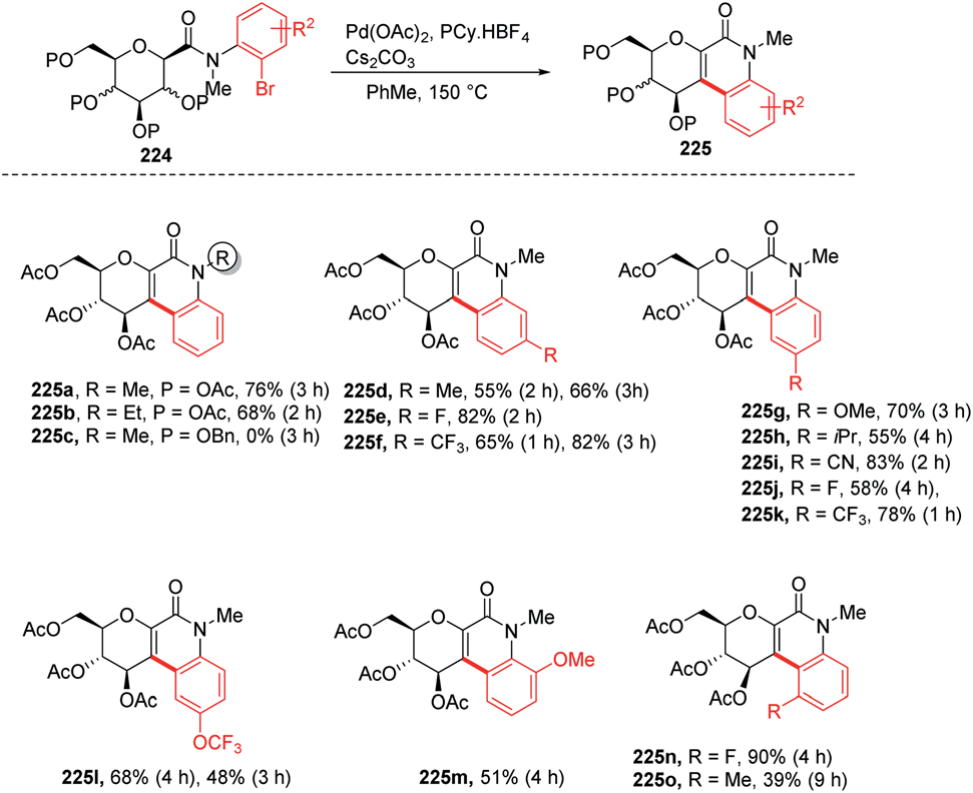
Intramolecular Pd-catalyzed anomeric C–H activation.

Over the past decade, C–H bond functionalization using transition metals emerged as an important strategy for the formation of carbon–carbon bonds. In 2017, Nicolas Probst and group reported^112^ a convenient intramolecular Pd-catalyzed anomeric C–H activation method for fused glycosylquinolin-2-ones and glycosylspirooxindoles synthesis from 2-bromophenyl glycosylcarboxamides (224) whose structures are close to that of the nitric oxide inhibitor of murine macrophage-like cells, *i.e.*, zanthodiolone and orixalone D. To determine the feasibility of the reaction, initial experiments were carried out under fixed conditions, with Pd(OAc)_2_ as the catalyst, toluene as the solvent, temperature of 150 °C and time of 3 h. Reacting 224 with ligand PCy_3_·HBF_4_, and base K_2_CO_3_ caused the partial conversion of the starting material and formed two new unexpected arylated compounds. On further screening and optimization, it was observed that the reaction efficiency was strongly affected by the base. Interestingly, the weak base was ineffective but on the other hand, a strong base like Cs_2_CO_3_ led to the total conversion of the starting material into the product with a 76% yield. Along with the base, the ligand is also responsible for the efficiency of the reaction. It was observed that this reaction was not limited to the β-glucosides but β-galactoside and β-mannosides were also capable of reacting under optimized conditions to give products (Scheme 58).

**Scheme 59:**
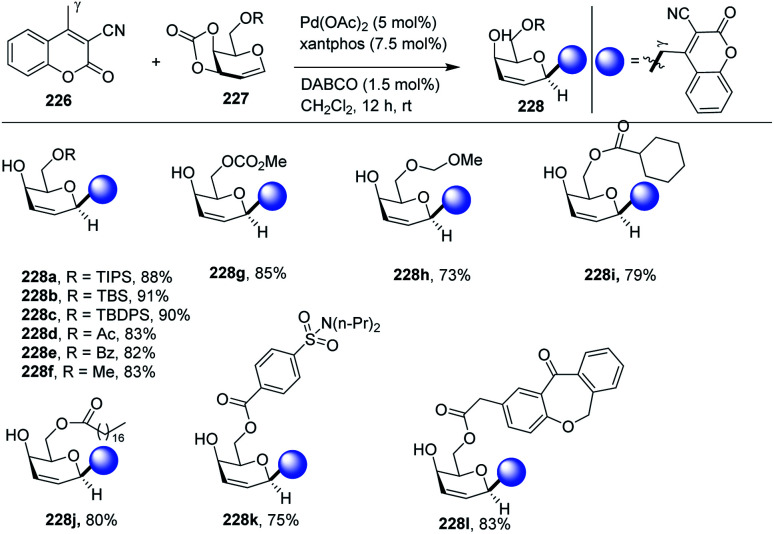
Pd-catalyzed vinylogous *C*-glycosylation.

Palladium-catalyzed regio- and stereoselective vinylogous *C*-glycosylation of α,β-unsaturated lactones (including coumarins) was achieved in high yields by Qiang Zhang and co-workers.^113^ The substrate scope of the reaction was carried out with a variety of substrates (51 examples) with high functional group tolerance. The substrate scope of the reaction was further extended with naturally and pharmaceutically derived substrates (228j–l). The practicality of this stereoselective vinylogous glycosylation approach was further enhanced with the gram-scale preparation of 2,3-unsaturated *C*-glycosides. A few selected examples are shown in Scheme 59.

## 4. Conclusions

In light of recent developments made in the synthesis of *C*-glycosides as inhibitors of glycosidases and glycosyltransferases, in natural product synthesis and their involvement in series of biological processes, *C*-glycosides have attracted the attention of organic chemists in recent decades. In this review, we have described the recent progress in the Pd-catalyzed transformations of carbohydrates into *C*-glycosides/branched sugars. It has been observed that different types of approaches have been used, such as cross-coupling, C–H activations, and directing group-assisted C–H bond activation, for the synthesis of *C*-glycosides/branched sugars. Despite having many literature reports, more atom economic and environmentally benign approaches are required since in most of the reactions preactivated substrates have been used instead of unactivated C–H bonds that could be used directly. Replacement of Pd catalysts with cheaper catalysts and ligand-free strategies further increases its applicability in a more cost-effective manner.

## Conflicts of interest

There are no conflicts to declare.

## Supplementary Material
